# The Groebke–Blackburn–Bienaymé reaction in its maturity: innovation and improvements since its 21st birthday (2019–2023)

**DOI:** 10.3762/bjoc.20.162

**Published:** 2024-08-01

**Authors:** Cristina Martini, Muhammad Idham Darussalam Mardjan, Andrea Basso

**Affiliations:** 1 Dipartimento di Chimica e Chimica Industriale, Università degli Studi di Genova, Via Dodecaneso 31, 16146 Genova, Italy; 2 Department of Chemistry, Universitas Gadjah Mada, Bulaksumur, Sekip Utara, Yogyakarta 55281, Indonesia

**Keywords:** Groebke–Blackburn–Bienaymé reaction, multicomponent reactions, heterocycles, imidazo[1,2-*a*]pyridines, isocyanides

## Abstract

The Groebke–Blackburn–Bienaymé (GBB) three-component reaction, discovered in 1998, is a very efficient strategy to assemble imidazo[1,2-*a*]-heterocycles starting from amidines, aldehydes and isocyanides. This review aims to exhaustively describe innovative aspects of this reaction achieved during the last five years, and classifies them into five categories: synthetic methods, building blocks, scaffolds, biological activities and physical properties.

## Introduction

The Groebke-Blackburn-Bienaymé (GBB) three-component reaction has been discovered in 1998 and during the course of the first two decades has emerged as one of the most exploited isocyanide-based MCRs, with more than 200 original publications reported and exhaustively reviewed by Boltjes and Dömling [1]. The GBB reaction turned 21 years old in 2019: the 21st birthday in many cultures is considered the attainment of maturity, and some traditions include that, upon reaching that age, the young person was given a key-pendant. The significance of this gesture was that the young person was considered old enough to be a key-holder to his family's home, and thus hold a symbolic 'senior' position in the family. Similarly, we can think of the GBB reaction in 2019 as having received the key-holder from the multicomponent reaction family, occupying now a senior position within it. The success encountered by the reaction until 2019 was far from being at its peak, in fact during the last 5 years about 70 new original works have been published, and it is therefore worth analyzing what are the new features of this, now, mature reaction.

**Scheme 1:**
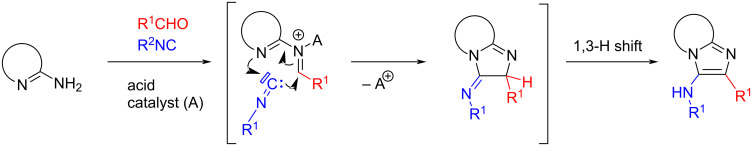
Mechanism of the GBB reaction.

Among isocyanide [2] based multicomponent reactions, the GBB reaction can be considered the third in importance after Ugi [3] and Passerini [4] ones, and, as the two venerable reactions, is an α-addition of an electrophile and a nucleophile to an isocyanide, followed by a suitable rearrangement, as depicted in Scheme 1.

Compared with the Passerini and Ugi reactions, however, GBB has different features, either advantageous or not. Certainly, the most negative aspect of the GBB reaction is that, compared with the others, it usually requires more drastic reaction conditions. For this reason, many studies have been carried out aimed at the discovery of new catalysts to allow the reaction to take place under the mildest possible conditions. This search goes in parallel with the urge to use increasingly diverse and complex building blocks, up to and including DNA conjugates, which would be degraded under the classical conditions developed by Groebke, Blackburn and Bienaymé.

On the other hand, the GBB reaction, compared with the Ugi and Passerini reactions, has an undeniable advantage, namely, the possibility of obtaining cyclic, aromatic, and drug-like compounds, rather than linear structures. The immediate consequence of this fact is that there are numerous studies on biological activities or photophysical properties (i.e., fluorescence) of GBB adducts.

Another advantage is that, by replacing the amidine component (classically 2-aminopyridines) with other heterocycles, different scaffolds are obtained, and this is extremely efficient in diversity-oriented synthesis (DOS) where a single methodology should generate diverse scaffolds.

This comprehensive review focuses on all these aspects, and has been divided into five chapters, describing, respectively: a) efforts to develop new and milder reaction conditions; b) the use of new building blocks; c) the generation of new scaffolds by coupling the GBB reaction with other transformations; d) the study of the biological and pharmacological properties of these fused heterocycles; e) the study of the photophysical properties of the GBB adducts. At the end of this overview, conclusions will be drawn about the future perspectives of this reaction.

## Review

### 1 Novel synthetic methods

The previously mentioned review, published in 2019 [1], had already highlighted how wide the variety of methods were used to conduct the GBB reaction: as much as 46 different catalytic systems had been reported in the literature at that time, along with some 30 solvents. The most widely used conditions, however, remained those originally discovered by Groebke, Blackburn and Bienaymé, namely the use of Sc(OTf)_3_, perchloric acid or *p*-toluenesulfonic acid as catalysts, and methanol, ethanol or toluene as solvents, or under solvent-free conditions. Although some new metal or Brønsted acid catalysts have been reported in the last few years, the main innovations can be found in the use of organic catalysts, enzymes, and compartmentations. A few reports on the in situ generation of reactants and on the reaction conducted under flow conditions can also be found.

#### 1.1 Metal and Brønsted acid catalysts

**Scheme 2:**
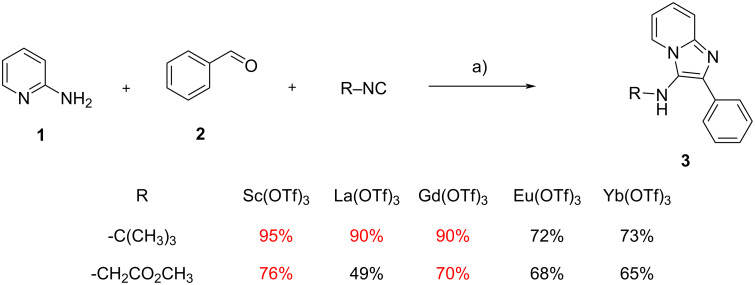
Comparison of the performance of Sc(OTf)_3_ with some RE(OTf)_3_ in a model GBB reaction. Conditions: a) M(OTf)_3_ (5 mol %), MeOH, 150 °C (MW, sealed tube), 0.5 h.

As previously mentioned, Sc triflate is the most widely used Lewis acid for the GBB reaction, generally exhibiting higher catalytic activity compared to other metal triflates, such as Yb, In or Bi. No extensive work had been done on rare earth (RE) triflates before the report by Longo et al*.* [5], who found La and Gd performing better than Eu and Yb, and similarly to Sc in a model reaction between 2-aminopyridine (**1**), benzaldehyde (**2**) and *tert*-butyl isocyanide to give **3** under microwave (MW) heating. However, when methyl isocyanoacetate replaced *tert*-butyl isocyanide, Gd(OTf)_3_ was superior to La(OTf)_3_, and again performed similarly to Sc(OTf)_3_ (Scheme 2). With these results in hand, Longo et al. evaluated the scope and limitations of this lanthanide, obtaining good to excellent results in the synthesis of 23 different GBB adducts, using aliphatic and aromatic aldehydes, both with electron-donating (ED) and electron-withdrawing (EW) substituents. It is worth mentioning that Gd triflate is much cheaper than scandium triflate, and that in this study the so called “gadolinium break” phenomenon [6], a discontinuity in the lanthanide properties, was not observed.

In 2016, Sashidhara et al. reported the catalytic effect of Ag(OTf) on GBB reactions, postulating its role in activating the attack of the isocyanides onto the imine intermediates [7]; some years later Liu et al. reported a similar role of AgOAc [8]. Although the reaction was tested on different substrates, the conditions were similar: 20 mol % of the catalyst, ethanol as solvent at 80 °C in the first case, 30 mol % of catalyst, ethylene glycol at 90 °C in the second case; however, a striking difference appears when the reaction was carried out in the absence of silver catalyst. Sashidhara et al. reported no conversion, while Liu et al. reported a 58% yield of the GBB product, postulating the ability of ethylene glycol to function as an activator (through hydrogen bonding) and a facilitator of proton transfer. After this discovery, ethylene glycol has never been employed in GBB reactions, if we exclude a 96-member library of GBB adducts reported very recently by Dömling et al. [9]. In this case, however, Sc(OTf)_3_ was used as the catalyst and the choice for ethylene glycol was dictated by the need to have a polar solvent with a high boiling temperature.

Shankar et al., however, reported that, by using hexafluoroisopropanol (HFIP) as the solvent, GBB adducts derived from glycal aldehydes could be isolated without additional catalysts in a few hours at 25–50 °C [10]. In this case, however, the role of the solvent as a Brønsted acid cannot be ruled out (this article will be discussed in more details in chapter 2).

**Scheme 3:**
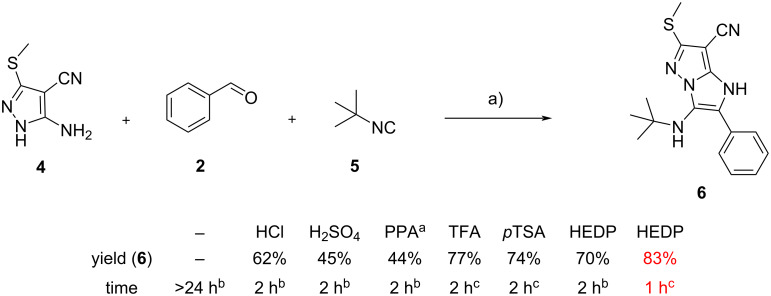
Comparison of the performance of various Brønsted acid catalysts in the synthesis of GBB adduct **6**. Conditions: a) catalyst (20 mol %), rt; ^a^pyrophosphoric acid; ^b^methanol as the solvent; ^c^methanol/water 1:1 as the solvent.

Another recent article on the use of Brønsted acids has been reported by Vilapara et al., who employed for the first time etidronic acid (1-hydroxyethane-1,1-diphosphonic acid, HEDP) as a green catalyst. Reactions were efficient at room temperature [11]; although the catalyst was tested only with 5-aminopyrazole **4**, and no comparison with a “classic” GBB reaction can be made, the authors compared HEDP with other catalysts on the same substrates, demonstrating that HEDP in MeOH/water was superior for yields, mildness, and less hazardous conditions (Scheme 3).

**Scheme 4:**
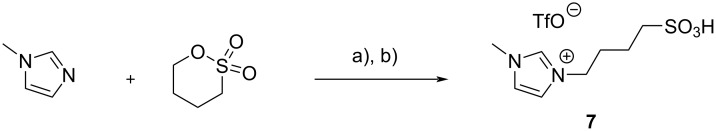
Synthesis of Brønsted acidic ionic liquid catalyst **7**. Conditions: a) neat, 60 °C, 24 h; b) TfOH, DCM, 0 °C to rt, 24 h.

Longo et al. prepared the Brønsted acid ionic liquid **7**, based on the 1-(butyl-4-sulfonic)-3-methylimidazolium cation, (Scheme 4) and tested it in the model reaction reported in Scheme 2 (R = *t-*Bu), obtaining a 71% yield using 20 mol % of catalyst in ethanol as the solvent under reflux conditions. The yield could be raised up to 83% when the reaction was heated in a MW oven at 150 °C [12]. With the optimized conditions, 22 GBB adducts were assembled with yields ranging from 42–93%. It is worth noting that the catalyst in this case was recovered and reused four times without appreciable loss of activity.

#### 1.2 Organic catalysts

**Scheme 5:**
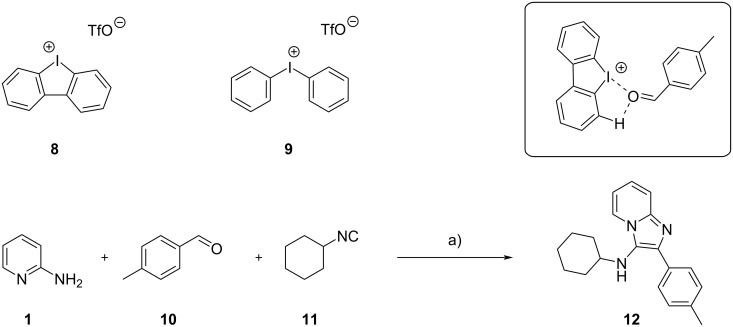
Aryliodonium derivatives as organic catalysts in the GBB reaction. In the box the proposed binding mode between catalyst **8** and *p*-tolualdehyde **10** is shown. Conditions: a) **8** (10 mol %), CHCl_3_/MeOH 95:5, 50 °C, 24 h.

Noncovalent organocatalysts display a few advantages compared to the traditional metal Lewis acids, such as lower environmental impact, higher stability to air and moisture, easier removal from the GBB products. In this regard, Bolotin et al. in 2022 have reported the high catalytic activity of diaryliodonium triflates such as **8** and **9** [13]. They studied a model reaction with 2-aminopyridine (**1**), *p*-tolualdehyde (**10**) and cyclohexyl isocyanide (**11**) both experimentally (by binding and kinetic studies) and theoretically (by density functional theory (DFT) calculations) (Scheme 5). Catalyst **8** was found to be significantly more active than **9**, owing to higher rigidity and the correct position of the *ortho* H atoms in close proximity to the σ-holes on the I atom. The authors demonstrated that hydrogen bonding between H in *ortho* position of **8** and both O atom of aldehyde and N atom of imine significantly increased the binding constants, leading to higher equilibrium concentrations of the electrophilically activated substrates. The H-bonds also increased the electrostatic potential on the σ-hole of the I atom, resulting in higher charge transfer values from the ligated species. Although no comparison was made with traditional metal catalysts, the authors synthetized 14 GBB adducts with yields ranging from 25% to 91% (compound **12** was obtained in 91% yield).

In the same year, the authors also reported aryl sulfonium and selenonium salts as alternatives to the previously described catalysts [14]. These compounds displayed an effective chalcogen bond donation to the substrates, in place of the halogen bonding previously described. Although their catalytic activity was reported to be lower than the one of aryl iodonium derivatives, this research contributed to the scarce number of publications on the catalytic activity of chalcogen-based noncovalent organocatalysts.

In 2023, Bolotin et al. published another article on the same subject [15], reporting a general improvement of electrophilic activation of carbonyl and imino groups by synergetic effect of aryl iodonium salts and silver cations. However, when similar conditions were tried in a GBB reaction, no cooperative effect was observed, but even a slight decrease in catalytic activity.

The use of thiamine hydrochloride as organic catalyst was reported by Yamajala et al., who were able to obtain the GBB adduct depicted in Scheme 2 (**3**, R = *t*-Bu) in 97% under solvent-free conditions at room temperature for 2 h [16]. Although thiamine had already been reported to be effective in other chemical transformations and its role in carbonyl activation in vivo through its thiazole ring is well known, no mechanism of action in the GBB condensation was proposed by the authors.

#### 1.3 Compartmented and enzyme-mediated reactions

Compartmentation of reaction media has already found many applications in chemistry. Amphiphilic molecules can associate in water to nanometer-sized micelles, above a certain critical concentration. Such micelles are characterized by a lipophilic core and a hydrophilic corona and can serve as heterogeneous systems for solubilizing hydrophobic chemicals in water. By concentrating reactants in nanometer-sized vessels, their reactivity is altered, and reaction rates are often accelerated. As a result, mild reaction conditions can be achieved.

**Scheme 6:**
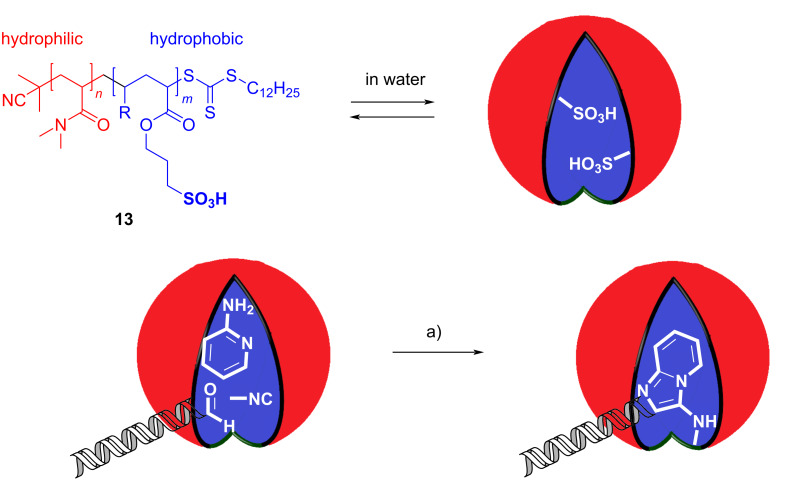
DNA-encoded GBB reaction in micelles made of amphiphilic polymer **13**. Conditions: a) **13** (50 equiv), water, 40 °C, 54 h.

Brunschweiger et al. employed the compartmentation strategy to overcome synthetic problems related to the preparation of a DNA-encoded GBB library [17]. DNA-encoded libraries (DELs) are widely used in screening projects, allowing the synthesis of a huge number of compounds as pools, and the identification of active ones by DNA sequencing. Great challenges, however, characterize the synthetic methodologies, since the chemistry must display a broad scope, be compatible with water and operationally simple, and preserve the genetic information (i.e., no harsh conditions, strongly acidic pHs, no oxidants or Lewis acids). In order to expand DELs to the GBB reaction, compartmentation was proposed to confine an acidic catalyst into the lipophilic core of micelles, inaccessible to water soluble DNA. Micelles were made of a sulfonic acid substituted, amphiphilic copolymer **13**. After reaction optimization, a narrow scope of 8 GBB adducts was obtained (Scheme 6).

Yamajala et al., in the last article discussed in the previous sub-chapter, also described the use of sodium dodecyl sulfate (SDS) as surfactant in GBB reactions performed in water, synthesizing 27 distinct imidazopyridines in 72–98% yield employing very mild conditions (10% SDS in water at rt with no added catalyst) [16].

Recently, the GBB reaction has also benefited from the use of inorganic nanocomposites and macromolecules, as demonstrated by the contributions of Rostamnia and Jung.

Rostamnia et al. intercalated, by ion exchange technique, Ni- based Keggin-type polyoxometalate α-[SiW_9_O_37_{Ni(H_2_O)}_3_] into a Zn_3_Al-based layered double hydroxide (LDH) [18]. Polyoxometalates are known for their tunable acidic (Brønsted/Lewis) properties, but also for their high solubility in polar solvents, therefore intercalation into a layered double hydroxide afforded a heterogeneous nanoreactor that could be employed in acid-catalyzed transformations. The GBB reactions were performed with 1 mol % catalyst at 35 °C under solvent-free conditions. A synergistic catalytic effect between polyoxometalate and LDH was evidenced by a higher catalytic activity of the composite compared to the individual constituents tested separately. Solvent-free conditions afforded better yields than traditional solvents (water, toluene, DCM, EtOH, MeOH) and 12 adducts were synthetized in 84–96% yield. Finally, the catalyst was recycled six times with a slight decrease in efficiency.

**Scheme 7:**
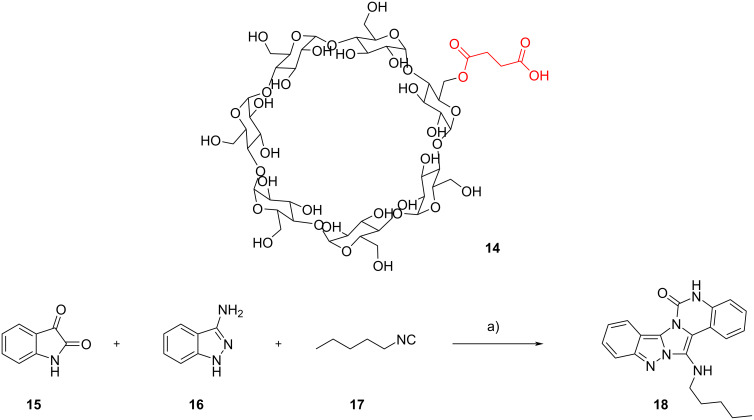
GBB reaction catalyzed by cyclodextrin derivative **14**. Conditions: a) **14** (1 mol %), water, 100 °C, 45 min.

Jung and Shinde, on the other hand, synthetized a supramolecular acid catalyst **14** combining β-cyclodextrins with succinic acid and tested it in a GBB reaction between isatin (**15**), indazol-3-amine (**16**) and pentyl isocyanide (**17**), yielding, after a ring expansion triggered by a retro-aza-ene reaction via a [1,5]-H shift [19], indazolo[3’,2’:2,3]imidazo[1,5-*c*]quinazolin-6(5*H*)-one **18** (Scheme 7) [20]. The favorable host–guest interaction between **14** and the reactants (demonstrated by 2D NMR and FTIR spectroscopy as well as by scanning electron micrography), combined with the acidity of the succinyl derivatization, allowed the obtainment of **18** in high yield under relatively mild conditions, and was extended to the synthesis of 23 analogues, all in high yields (>86%). The catalyst could be recovered from the reaction medium by precipitation and reused up to five times without loss in activity.

**Scheme 8:**
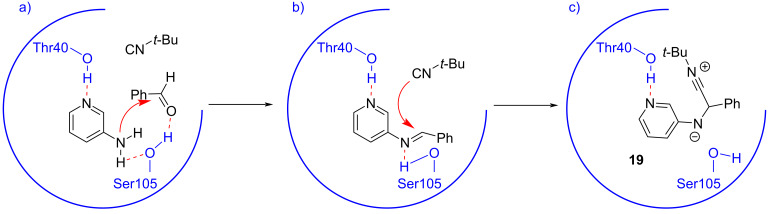
Proposed mode of activation of CALB. a) activation of the substrates; b) activation of the imine; c) addition of the isocyanide.

In 2020, Tyagi et al. reported the first biocatalytic GBB reaction using lipase [21]. The model reaction depicted in Scheme 2 (R = *t*-Bu) was tested in ethanol at room temperature with various enzymes, where *Candida antarctica* lipase B (CALB) and *Aspergillus niger* gave best results, affording **3** in 63% and 64% yields, respectively. For further investigations, however, CALB was chosen due to its lower cost and easier availability. Optimization of enzyme loading and substrate ratios increased the yield up to 91%. Immobilization of the enzyme on silica particles was not detrimental to the yield, but allowed enzyme recycling, albeit with a slow but continuous deterioration of catalytic activity. Preliminary molecular docking and molecular dynamics simulation studies revealed that Thr40 and Ser105 residues played a crucial role in catalyzing the GBB reaction, forming hydrogen bonds with 2-aminopyridine substrate, increasing its nucleophilicity and improving its orientation. Furthermore, Ser105 formed a strong hydrogen bond also with benzaldehyde, making it a better electron acceptor. Interestingly, also the imine intermediate showed strong interaction with Thr40 and Ser105 residues, so becoming a good electrophile for the addition of *tert*-butyl isocyanide. On the other hand, the addition of *tert*-butyl isocyanide on the imine altered the orientation of adduct **19**, suppressing the interaction with Ser105 (Scheme 8).

**Scheme 9:**
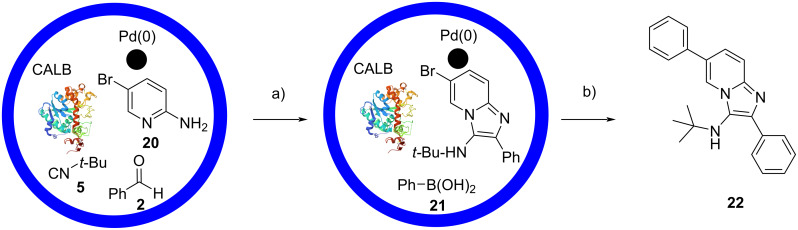
One-pot GBB reaction–Suzuki coupling with a bifunctional hybrid biocatalyst. Conditions: a) Pd(0)-CALB@SiO_2_, EtOH, rt; b) K_2_CO_3_, dioxane, 60 °C. The crystal structure of CALB from *Candida antarctica* (4K6G, https://doi.org/10.2210%2Fpdb4K6G%2Fpdb%29 was taken from RCSB Protein Data Bank [23].

Very recently, Tyagi et al. reported the double encapsulation of CALB and Pd(PPh_3_)_4_ within silica, obtaining an enzyme-metal biohybrid catalyst [22]. The authors tested it in a one-pot GBB reaction–Suzuki coupling, employing 5-bromo-2-aminopyridine (**20**), benzaldehyde (**2**), *tert*-butyl isocyanide (**5**) and phenylboronic acid (**21**). Compound **22** was obtained in 87% yield by performing the GBB reaction at room temperature and then, upon addition of the boronic acid, heating at 60 °C (Scheme 9). Similar efficiencies were observed with a wide range of boronic acids (10 examples, 49–87% yield). The biohybrid was suitable also for gram-scale synthesis and for storage at room temperature under inert atmosphere, showing no loss of activity after 30 days. On the other hand, reusability seemed problematic, as significant decrement in yield was observed after the second cycle.

Kinetic target-guided synthesis (KTGS) has emerged as a promising strategy in drug discovery [24]. It relies on a drug target serving as a template, selecting the ligand building blocks with highest affinity and bringing them in proximity within the binding site. Building blocks are opportunely chosen to have complementary reactivity and therefore to react irreversibly within the binding site and assemble into the final ligand. Before the work described below, KTGS was applied to multicomponent reactions in limited cases [25]. Van der Veken et al. selected as template the protein urokinase plasminogen activator, a serine protease targeted in oncology and inhibited by imidazopyridines [26]. The main challenges of this project were the compatibility of the GBB reaction with typical KTGS conditions, such as aqueous solvent at near-physiological pH and high dilution, and the achievement of selective ligand amplification. First, molecular modeling studies were carried out to verify that building blocks and intermediates could bind the target protein in a manner comparable to the binding mode of known imidazopyridine inhibitors. Second, the possibility that reactants could covalently modify the enzyme was ruled out by analysis of the relevant literature. Finally, appropriate building blocks displaying additional functionalities capable of interactions with the target were synthetized. After optimization of the reaction medium and of the concentration of reactants, and after setting up an analytical method relying on UPLC with tandem quadrupole detector, the on-target KTGS experiments were conducted. In a first set of experiments, a single combination of reactants was studied, but a major hurdle was found to be the imine formation step in aqueous media. Consequently, imines were first prepared as metastable adducts (with benzotriazole or *p*-TSA as stabilizers) and then tested with isocyanides in the presence of the template protein, involving either a single combination of building blocks or competition between two of them. In both attempts, however, the results were not in line with expectations, as the enzyme proved to be unable to guide the synthesis of GBB adducts. The authors concluded, however, that this study, especially for the use of metastable imines, could serve as a starting point for the development of other TGS projects involving imine-based multicomponent reactions.

#### 1.4 In situ generation of reactants and use of enabling technologies

Despite their high versatility, isocyanides have some drawbacks, such as their repellent smell (but only for most of unhindered small ones), their partial instability and their potential toxicity [27], thus in situ generation represents a sustainable alternative for their conventional use and this issue has been recently reviewed by Baht and Heravi [28]. In situ generation of isocyanides has been applied also to the GBB reaction in the past, as reported by Guchhait et al. in 2013 [29] and Dömling et al*.* in 2015 [30], however, in both cases an external acid was required for the multicomponent reaction to occur. The recent report by Salunke et al., instead, exploits the presence of in situ generated hydrogen iodide, deriving from the mixture of I_2_, PPh_3_ and Et_3_N used for the dehydration of the formamide into isocyanide [31]. All substances are mixed from the very beginning, and the reaction could be carried out at room temperature for 12 h, or heated under MW at 60 °C for 30 min. Although this approach uses large excess of reagents and produces a lot of waste, the intrinsic operational simplicity and the reduced reaction times allowed its use in teaching labs at the undergraduate level. This represents one of the first examples of MCR demonstration for educational purposes [32].

Also, the aldehydic component has been generated in situ through Fe_3_O_4_-mediated aerobic oxidation of benzyl alcohols. Magnetic nanoparticles were supported by Shaabani and Farhid on spent coffee ground and served also as catalyst for the GBB reaction, although the role of coffee ground was not clearly explained [33].

In 2021, Poole et al. [34] reported the first GBB reaction performed under flow conditions. The method proved to be robust, efficient and scalable, and showed a broad functional group tolerance. A commercial apparatus was used and two stock solutions (the first consisting of the amines (1 equiv), the aldehydes (2 equiv) and mineral acid (HCl, 0.1 equiv) dissolved in ethanol, the second made of the isocyanides (2 equiv) dissolved in the same solvent) were pumped into the coil heated at 130 °C. Residence time was 50 min and the isolated yields (27 examples) ranged from 33% to 90%; noteworthy, the reaction performed under flow conditions afforded the desired product also when the corresponding batch reaction failed. The authors provided no comment on whether the imine could already be formed in the stock solution before pumping into the reaction coil. A multigram synthesis was also performed, affording a GBB adduct in more than 4 grams in a total time of 8 hours. The same amount was obtained in batch in a total time of 20 hours.

### 2 Novel building blocks

The scope and limitations of the GBB-3CR have been accurately delineated up to 2018, outlining a comprehensive understanding of its development, observing different combinations of starting materials and elucidating the effect of substituents on each of the partners [1].

Since 2019, research has been focused on broadening its scope with the aim of moving towards a more sustainable chemistry or to impart specific properties to the final products, therefore a series of novel building blocks, mainly carbonyl derivatives, have been used as starting materials.

#### 2.1 Novel aldehydes

Carbonyl compounds are among the most common starting materials for multicomponent reactions: aldehydes play a central role also in the GBB-3CR. Several experiments have demonstrated the tolerability of a wide range of aldehydes, starting from one of the most used, benzaldehyde, passing through heteroaromatic and aliphatic structures with different substituents.

In this context, some examples where the aldehyde functionality is incorporated into sugar derivatives, natural compounds or molecules derived from biomass have been recently published. Due to the relatively drastic conditions of the GBB reaction and the inherent lability of these structurally complex building blocks, often specific reaction conditions have been developed to successfully obtain the multicomponent adducts.

**Scheme 10:**
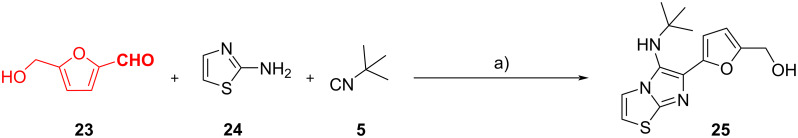
GBB reaction employing 5-HMF (**23**) as carbonyl component. Conditions: a) TFA (20 mol %), EtOH, 60 °C, 2 h.

An intriguing study is reported by Porcal et al. [35] in which they evaluate the use of 5-hydroxymethylfurfural (5-HMF, **23**). Nowadays, the need to move towards more sustainable protocols is impending and the emphasis on green chemistry and on the use of renewable carbon sources is huge. Indeed, the use of 5-HMF (**23**) [36], a renewable carbon source derived from biomass, responds to these requirements. This aldehyde displays additional functionalities useful for further modifications of the GBB adducts, but some optimization was necessary due to its intolerance to high temperatures and reaction conditions. The authors performed the optimization study using 5-HMF (**23**), 2-aminothiazole (**24**) and *tert*-butyl isocyanide (**5**, Scheme 10).

Inspired by a procedure reported by Demjén et al. [37], they found the best conditions using EtOH as a green solvent, trifluoroacetic acid (TFA, 20 mol %) or Yb(OTf)_3_ (5 mol %) as acidic catalyst, and a non-microwave instant heating reactor, performing the reaction at 60 °C and obtaining **25** in 78% yield. Moving forward to investigate the scope of the reaction with different 2-aminoamidines and isocyanides, they obtained 18 different products with isolated yields up to 87%.

**Scheme 11:**
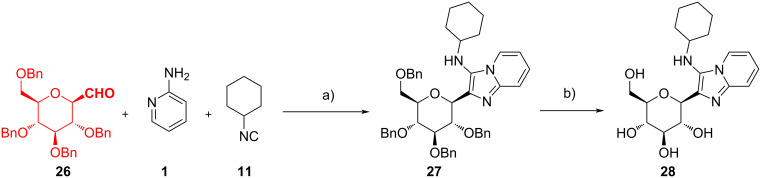
GBB reaction with β-C-glucopyranosyl aldehyde **26**. Conditions: a) InCl_3_ (20 mol %), MeOH, 70 °C, 2–3 h; b) Pd-C/H_2_, MeOH, rt.

The use of sugar-derived building blocks has been reported by various authors. Prasad et al. studied the synthesis of 2-(β-ᴅ-glucopyranosyl)-3-*N*-alkylamino-1-azaindolizines **28** to get novel bioconjugates [38] and of 5-(3”-alkyl/arylamino-1”-azaindolizin-2”-yl)-2’-deoxyuridines **31** to obtain new fluorescent products [39]. Starting from perbenzylated β-C-glucopyranosyl aldehyde **26**, 2-aminopyridine (**1**) and cyclohexyl isocyanide (**11**), they synthesized C-glucoside **28** (Scheme 11). β-C-Glucopyranosyl aldehyde **26** was obtained from ᴅ-glucose following a procedure already established [40]. Aware of the trials made by Manvar et al. [41] with catalytic amount of acetic acid, the authors initially obtained **27** in moderate yield. Subsequently, testing different catalysts and solvents, they found out that performing the reaction in the presence of InCl_3_ (20 mol %) in MeOH at 70 °C for 2–3 h was the best option (72%). With the optimized conditions in hand, they used different 2-aminopyridines and alkyl isocyanides, getting 14 distinct products with 56–88% yield. The reaction did not work with 2-aminopyridines with electron-withdrawing groups, and this was attributed to their reduced nucleophilicity.

**Scheme 12:**
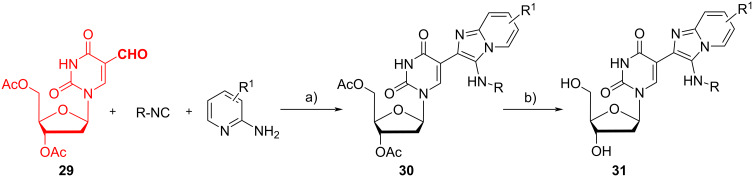
GBB reaction with diacetylated 5-formyldeoxyuridine **29**, followed by deacetylation of GBB adduct **30**. Conditions: a) TFA (5 mol %), MeOH, 60 °C; b) K_2_CO_3_, MeOH/H_2_O, 25 °C.

In their second work about this topic, Prasad et al. synthesized 5-(3”-alkyl/arylamino-1”-azaindolizin-2”-yl)-2’-deoxyuridines **31** as new base-modified fluorescent nucleosides with high Stokes’ shift, potentially useful for investigating nucleic acid structure and functions. The authors synthesized deoxyuridine-based aldehyde **29** starting from thymidine in 30% yield, according to a known procedure [42]. Then, they tested two different strategies: in the best one (depicted in Scheme 12) **29** was used in the GBB-3CR, then adduct **30** was deacetylated to afford the final product **31** in an overall yield of 83–95%. The second strategy, based on the deacetylation of **29** followed by the GBB-3CR, led to a lower overall yield of 21–23%. The reaction with different 2-aminopyridines and alkyl/aryl isocyanides afforded the intermediates **30** in 86 to 96% yield, and the subsequent deacetylation with potassium carbonate was almost quantitative (97–99% yield). It is worth mentioning that the GBB reaction starting from the deacetylated substrate also leads to good results (83–91% of yield), but the overall yield is lowered due to a sluggish deacetylation reaction of **29**.

**Scheme 13:**
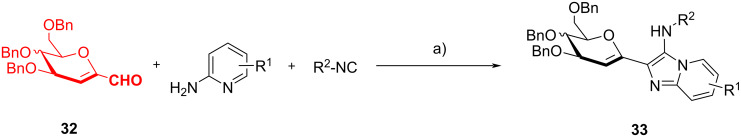
GBB reaction with glycal aldehydes **32**. Conditions: a) HFIP, 25 °C, 2–4 h.

Sugar-based aldehydes were employed also in the work reported by Shankar et al. [10], already mentioned in chapter 1. The authors established a solvent-catalyzed GBB-3CR to synthesize glycosylated imidazo[1,2-*a*]pyridines **33** starting from 1-formyl glycals **32**; using HFIP as the solvent, the addition of any metal catalyst was not needed (Scheme 13). Glycal aldehydes **32** were synthetized starting from ᴅ-glucose and ᴅ-galactose, following an established procedure [40]. The scope of the reaction was evaluated using differently substituted amidines, and 9 different 2-(β-ᴅ-glycal-1-yl-)-3-*N*-alkylamino-1-azaindolizines **33** were synthesized in excellent yields (71–89%).

Dömling et al. have used glyoxal dimethyl acetal as orthogonal bifunctional monoprotected aldehyde to synthetize GBB dimers as potential fluorophores. More details are given in chapter 5 [43].

**Scheme 14:**
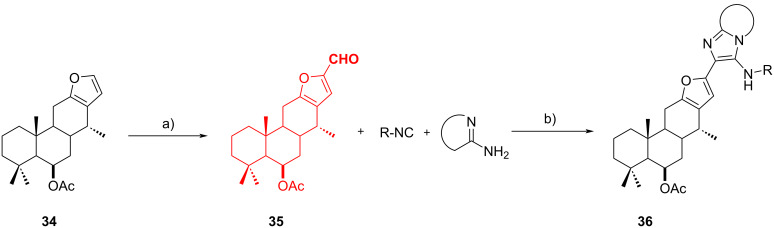
Vilsmeier–Haack formylation of 6-β-acetoxyvouacapane (**34**) and subsequent GBB reaction. Conditions: a) POCl_3_, DMF, 0 °C to rt; b) Sc(OTf_3_) (10 mol %), DCM/MeOH, rt.

The GBB reaction can be exploited also in the late-stage functionalization of natural molecules, with the possibility of generating pseudo-natural compound libraries: Cortés-García et al. [44] applied this strategy to vouacapane. The authors developed a two-step reaction for the synthesis of fused vouacapane-azoles **36**. Starting from the 6-β-acetoxyvouacapane (**34**), a natural product isolated from the dichloromethane extracts of the leaves of *Caesalpinia platyloba* by column chromatography, they performed a Vilsmeier–Haack formylation at the furan ring (Scheme 14) obtaining aldehyde **35** in 92% yield. This, in turn, was used as starting material in a GBB-3CR performed at room temperature to avoid opening of the furan ring. The authors explored the substrate scope by using different isocyanides and 2-aminoazines, obtaining 6 potentially bioactive pseudo natural products **35** in low to moderate yield (21–67%).

**Scheme 15:**
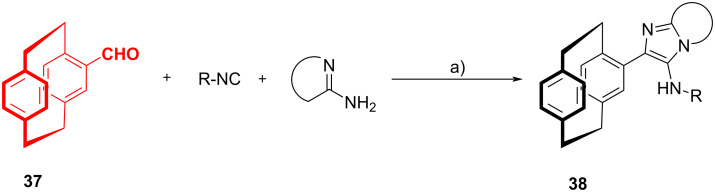
GBB reaction of 4-formlyl-PCP **37**. Conditions: a) HOAc or HClO_4_, MeOH/DCM (2:3), rt, 3 d.

Bräse et al. [45] reported the possibility of exploiting the GBB-3CR to synthesize a cyclophanyl-imidazole-based library of ligands. The synthesis of ligands based on the [2.2]paracyclophane (PCP) moiety, thanks to its structural features and inherent planar chirality upon selective substitution, has been recently reviewed by the same author [46]. Starting from 4-formylcyclophane **37**, a GBB-3CR with different isocyanides and amidines was exploited to synthesize PCP-based imidazo[1,2-*a*]pyridyl-substituted ligands **38** (Scheme 15). During the study of the scope, it was observed that 2-aminopyrazines afforded lower yields (12–48%) than 2-aminopyridines (42–87%), and this was attributed to their reduced nucleophilicity. Also the synthesis of a bis-imidazo[1,2-*a*]pyridine scaffold was achieved in 43% yield from the pseudo-*para*-substituted bis-aldehyde, using the same conditions, but longer reactions times (6 days). Following the same approach, the authors also developed a one-pot protocol for a GBB-3CR through an in situ generation of the cyclophanyl isocyanide. They synthesized the formamido[2.2]paracyclophane, and then, following the procedure reported by Dömling [30], dehydrated the formamide in situ and reacted the resulting isocyanide with 2-aminopyridine and an aldehyde. As a proof of concept, one of the GBB adducts was transformed into a *N*,*C*-palladacycle and was investigated as a potential catalyst for carbon–carbon bond-forming reactions. Furthermore, the authors studied in detail the fluorescence properties of the GBB adducts, that will be discussed in chapter 5.

**Scheme 16:**
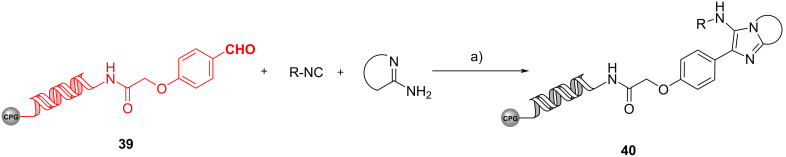
GBB reaction with HexT-aldehyde **39**. Conditions: a) **39** (20 nmol) and amidine (20 μmol), MeOH, rt, 6 h, then isocyanide (20 μmol) and AcOH (1 mol %), rt, 16 h.

As already discussed in section 1.3, Brunschweiger et al. [47] reported the synthesis of DNA–imidazole heterocyclic conjugates by the GBB-3CR. An alternative to the previously discussed encapsulated solution-phase synthesis is the solid-phase approach in which the DNA barcodes are synthesized on CPG (controlled pore glass). In this study they used a benzaldehyde unit conjugated to a hexathymidine oligonucleotide **39** and reacted it with various isocyanides and amidines to obtain a small collection (9 examples) of adducts **40** in yields ranging between 23% and 95%; large excess of reagent and a weak Brønsted acid were essential for the success of the reaction (Scheme 16). A cheminformatic analysis revealed that GBB-based encoded libraries could cover a diverse chemical space, different from the one covered by encoded libraries obtained with other isocyanide-based MCRs.

#### 2.2 Novel heterocyclic amidines

**Scheme 17:**
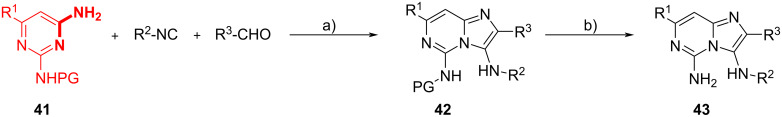
GBB reaction of 2,4-diaminopirimidine **41**. Conditions: a) Sc(OTf)_3_ (20 mol %), MeCN, 120 °C (MW), 1 h; b) TFA, reflux, overnight.

Almost 90 different amidines had already been tested until 2018 [1], however, room for novel building blocks or old ones with novel reactivity is still available as demonstrated for example by Dömling et al. [48]. They focused on the use of 2,4-diaminopyrimidines **41** to synthetize diaminoimidazopyrimidines **43**. This substrate was already employed by Lavilla et al. [49] to obtain selectively imidazo[1,2-*a*]pyrimidin-7-amine derivatives, due to the higher reactivity of the amine in position 2. To uncover the reactivity of the amine in position 4, and thus obtain imidazo[1,2-*c*]pyrimidin-5-amines **43** instead, the authors synthesized a series of 2-amino-protected compound **41** through an aromatic nucleophilic substitution on 2-chloropyrimidin-4-amines. Derivatives **41** were then reacted under the optimized conditions, leading to the formation of a series of GBB adducts **42**, finally deprotected with TFA to get **43** in an overall yield of 40–80% (Scheme 17). In this study, 14 distinct products were obtained, and the only limitation was observed using aliphatic aldehydes, due to the instability of the deprotected GBB adducts. Imidazopirimidines are a privileged scaffold incorporated in many bioactive compounds.

**Scheme 18:**
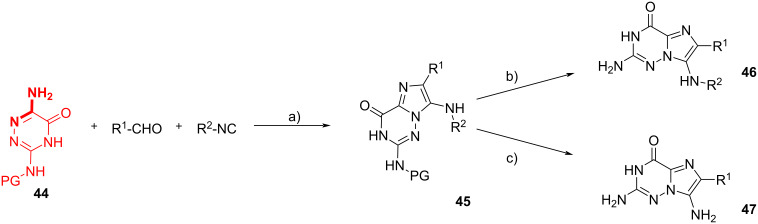
Synthesis of *N*-edited guanine derivatives from 3,6-diamine-1,2,4-triazin-5-one **44**. Conditions: a) Sc(OTf)_3_, MeOH, 100 °C; b) **45** (0.2–0.5 mmol), 0.1 M TFA, 80 °C, 12 h; c) **45** (0.1–0.5 mmol), 0.1 M TfOH, 55 °C, 4 h.

Dömling et al. [50] also reported the synthesis of *N*-edited guanine derivatives. Different drugs display the guanine motif, fundamental for its biological activity is a triad HBA–HBD–HBD (HBA = hydrogen bond acceptor, HBD = hydrogen bond donor) included in its structure. The authors propose a one-pot two-step procedure, combining the GBB-3CR and an acid-assisted deprotection reaction, to get a library of imidazo[2,1-*f*][1,2,4]triazin-4(3*H*)-ones **46**, characterized by a flat heterocyclic ring system displaying the essential triad. Guanine derivatives are typically synthesized through a sequential multistep procedure, however, by employing 3,6-diamino-1,2,4-triazin-5-one **44** as amidine derivative, **46** could be simply obtained through a GBB-3CR. The amino group in position 3 was opportunely protected to drive the GBB reaction to the selective formation of **45**. Microwave heating appeared to be beneficial compared to traditional heating for the outcome of the reaction (60% yield vs 49%). A consistent library of 22 compounds was prepared, in yields ranging from 21 to 75%, and the GBB adducts were finally deprotected under acid conditions. When TFA was used partial deprotection to **46** was achieved, while the use of triflic acid (TfOH) resulted also in the cleavage of the alkyl group derived from the isocyanide, with formation of **47** (Scheme 18).

**Scheme 19:**
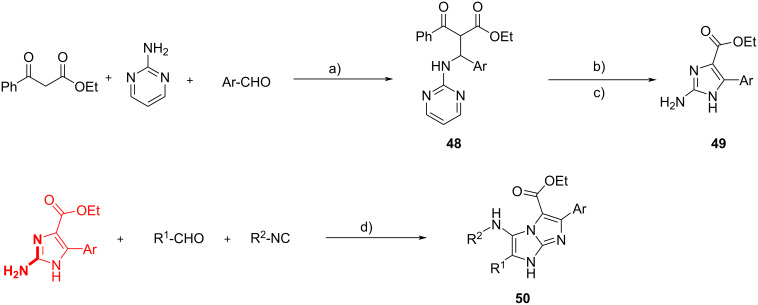
Synthesis of 2-aminoimidazoles **49** by a Mannich-3CR followed by a one-pot intramolecular oxidative annulation/ring cleavage and subsequent GBB-3CR. Conditions: a) phosphotungstic acid (PTA) or TMSCl, MeCN or H_2_O, 60 or 80 °C; b) IBX, IPT, DMA, 80 °C, 30 min; c) NH_2_OH∙HCl/Na_2_CO_3_, DMA, 50 °C, 16 h; d) HClO_4_ (20 mol %), MeCN, 60 °C, 24 h.

Kanizsai et al*.* [51] have reported the synthesis of 4,5-disubstituted 2-amino-1*H*-imidazoles and their further modification through the GBB-3CR. The 2-aminoimidazole moiety can be found in different alkaloids isolated from sponges and exhibits useful bioactive properties. The methodology employed by the authors to assemble C4/C5-functionalized 2-aminoimidazoles **49** exploited a Mannich condensation followed by an iodoxybenzoic acid (IBX) and *N*-iodophthalimide (IPT)-mediated intramolecular oxidative annulation and a hydroxylamine-induced ring cleavage of intermediate **48**. With this one-pot sequential procedure they synthesized **49** in yields up to 95% (Scheme 19).

The subsequent GBB-3CR led to the unique 1*H*-imidazo[1,2-*a*]imidazole core **50** with 4 distinct diversification points. The scope of the reaction was limited: alkyl aldehydes and aryl/benzyl isocyanides could not be used, and 4 distinct compounds were obtained in yields of 23–40%. Nevertheless, these results represent an improvement compared to previous studies where substitution on C4 of the 2-aminoimidazole scaffold prevented the GBB reaction to occur [52].

**Scheme 20:**
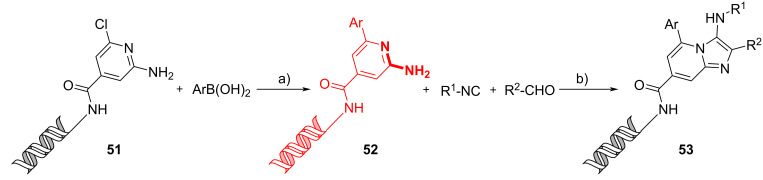
On DNA Suzuki–Miyaura reaction followed by GBB reaction. Conditions: a) CsOH, sSPhos-Pd-G2; b) AcOH (30 equiv), H_2_O/DMSO, 25 °C, 24 h.

As it has already been described, DNA encoded GBB adducts can be effectively used in DEL screening techniques. Hwang et al. incorporated 2-amino-6-chloropyrimidine-4-carboxylic acid into a DNA sequence, reacted the resulting conjugate **51** through a Suzuki–Miyaura reaction and then subjected the adduct **52** to a GBB reaction with various aldehydes and isocyanides (Scheme 20) [53]. Focusing on the GBB part, the authors tested both Lewis acids (Sc(OTf)_3_ or Yb(OTf)_3_) and Brønsted acids (NH_4_Cl or AcOH) and found that the best results could be obtained using 30 equiv of AcOH in DMA, with no damage of DNA observed. The GBB reaction, under these mild conditions, demonstrated a wide range of scope. Different aldehydes were tested: benzaldehydes with different substituents, heteroaromatic and bicyclic aromatic aldehydes, all giving good results. Only aliphatic aldehydes showed a higher variability (50–98%) and in some cases also the classic Ugi adducts were observed. Regarding the isocyanide, excellent yields were achieved for all tested reagents, such as aromatic, aliphatic, and sterically hindered ones. Problems were only observed using a morpholine derivative because the base properties of the reagent could hinder AcOH from catalyzing the reaction.

**Scheme 21:**
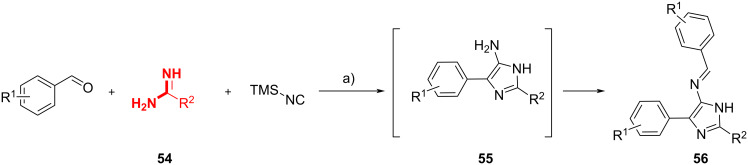
One-pot cascade synthesis of 5-iminoimidazoles. Conditions: a) Na_2_SO_4_, DMF, 220 °C (MW).

Smith et al. [54] explored the potential of acyclic amidines **54** in the synthesis of substituted-imidazoles via the GBB reaction under MW heating (Scheme 21). The resulting 5-aminoimidazoles **55** were in situ-reacted with the aldehydes to yield 5-iminoimidazoles **56**. When benzamidine (**54**, R^2^ = Ph) was used, aldehydes bearing electron-rich groups gave the products in higher yields compared to those with electron-poor groups. This study demonstrated that the GBB reaction could take place when acyclic amidines containing an aromatic ring or heteroatoms (**54**, R^2^ = NHAc, NH_2_) were used, while aliphatic amidines (**54**, R^2^ = H, CH_3_, CF_3_) were unsuccessful, indicating that the resonance stabilization of the amidine was crucial to the reaction.

**Scheme 22:**
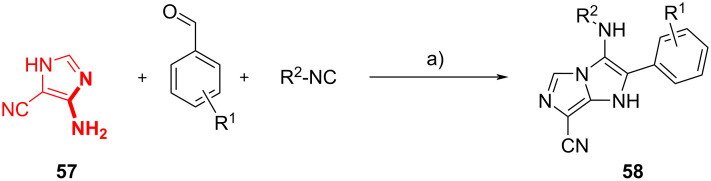
GBB reaction of 5-amino-1*H*-imidazole-4-carbonile **57**. Conditions: a) HClO_4_ (5 mol %), MeOH, rt, 24 h.

Finally, Guillaumet et al. [55] have proposed a simple way to synthesize the 2-substituted 1*H*-imidazo[1,5-*a*]imidazole scaffold. This scaffold has a significant relevance in drug synthesis and was not deeply studied yet. As depicted in Scheme 22, 5-amino-1*H*-imidazole-4-carbonitrile (**57**) was used as heterocyclic amidine, to obtain the desired bicyclic adducts **58**. The solvent choice was limited to methanol due to solubility problems, and HClO_4_ was selected because other Brønsted acids caused amine deprotection. The GBB adducts **58** could be further elaborated through a Buchwald intramolecular nucleophilic substitution/cyclization, as it will be described in section 3.3.

### 3 Novel scaffolds

Owing to the flexibility for variation of the GBB reaction, it has been widely employed in combination with other reactions for the formation of novel scaffolds with high level of complexity. The general approach to generate complex architectures is the installation of functional groups on the GBB precursors which can be involved in further modifications. In addition, the GBB-like reactions can also be used to deliver other heterocyclic motifs.

#### 3.1 One-pot synthesis

As an efficient approach in organic synthesis, one-pot synthesis has been exploited in the post-modification of GBB products. This approach offers several advantages since multiple bond formation, synthetic transformation and heterocyclic construction can be conducted in a single reactor [56]. Novel scaffolds derived from the GBB products have been successfully constructed through three types of one-pot synthesis, namely cascade/domino reaction, one-pot stepwise synthesis and multicomponent reaction.

In the one-pot cascade reaction, the complexity of heterocycles was achieved due to the presence of functional groups formed in the GBB reaction which allow further reactions to occur. In the case of one-pot stepwise synthesis, the GBB products were introduced to the subsequent reactions in the same pot without isolating them from the reaction mixture. In the one-pot multicomponent reaction, three substrates with di- or tri-functional groups were engaged in the GBB reaction to produce the heterocyclic polymers.

**Scheme 23:**
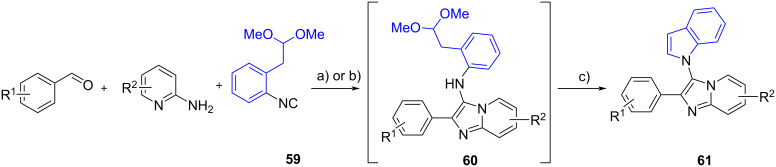
One-pot cascade synthesis of indole-imidazo[1,2,*a*]pyridine hybrids. In blue the structural motif involved in the secondary transformation. Conditions: a) TFA (20 mol %), 1,4-dioxane, 110 °C, 2 h (Panda and Ganesher [57]); or b) ZnCl_2_ (5 mol %), 1,4-dioxane, reflux, 5–6 h, c) DCM/TFA (1:1), rt, 4–5 h (Jain and Sen [58]).

Panda and Ganesher [57] reported the one-pot cascade synthesis of indole-imidazo[1,2,*a*]pyridine hybrids **61** (Scheme 23). In this study, the convertible isocyanide 1-isocyano-2-(2,2-dimethoxyethyl)benzene (**59**, Kobayashi–Wessjohann isocyanide) was utilized as one of the precursors in the GBB reaction. The acetal-substituted products **60** underwent TFA-promoted deprotection which triggered the intramolecular cyclization to furnish the indole moiety in the desired products **61**. A control experiment showed that the GBB product **60** was obtained as the sole product in the absence of acid catalyst.

Another group developed the same reaction via a two-step synthesis [58]. Initially, the three starting materials were subjected to the GBB reaction in the presence of a catalytic amount of ZnCl_2_ to give the fused products **60**. Then, the cyclization was carried out using TFA to deliver **61** (14 examples) in yields ranging from 60% to 65%.

**Scheme 24:**
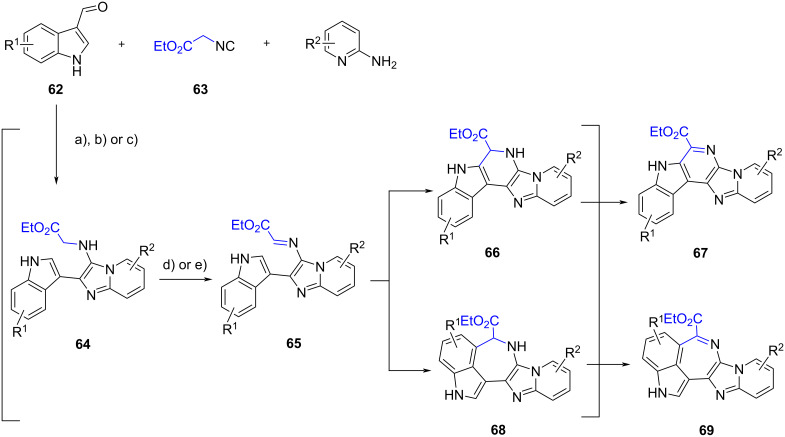
One-pot cascade synthesis of fused polycyclic indoles **67** or **69** from indole-3-carbaldehyde. Conditions: a) Yb(OTf)_3_ (20 mol %), O_2_ (air), MeCN, 80 °C, 22 h, b) Yb(OTf)_3_ (20 mol %), CoBr_2_ (5 mol %), O_2_ (air), MeCN, 80 °C, 22 h; c) Yb(OTf)_3_ (20 mol %), MeCN, 80 °C (MW), 1 h; d) CoBr_2_ (5 mol %), O_2_ (air), MeCN, 80 °C, 22–60 h; e) I_2_, MeCN, 80 °C, 48 h.

**Scheme 25:**
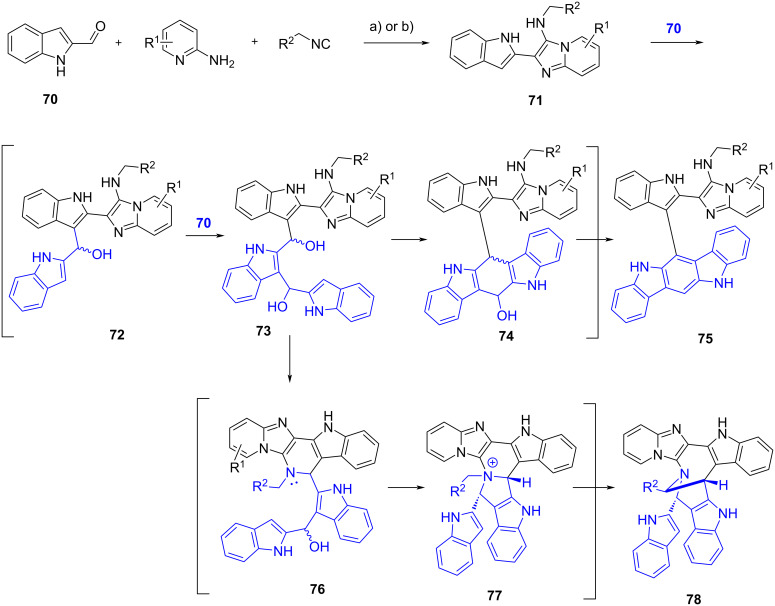
One-pot cascade synthesis of linked- and bridged polycyclic indoles from indole-2-carbaldehyde (**70**). Conditions: a) for the synthesis of **75**: Yb(OTf)_3_ (20 mol %), MeCN, rt, 48–72 h; b) for the synthesis of **78**: Yb(OTf)_3_ (20 mol %), MeCN, 110 °C (MW), 90 min.

Lavilla et al. [59] explored the reactivity of indole carbaldehydes in the extended GBB multicomponent reaction. In this study, various fused-, linked- and bridged polyheterocycles were generated through different reaction pathways, depending on the type of indole carbaldehydes and the reaction conditions. The study was started by reacting 2-aminopyridines, indole-3-carbaldehyde **62** and ethyl isocyanoacetate (**63**) under open air in the presence of Yb(OTf)_3_ catalyst (Scheme 24, conditions a). The results showed that fused polycyclic indoles **67** were obtained (albeit in lower yields, 25%) instead of the corresponding GBB products **64**. The authors proposed that adducts **64** were transformed into the intermediates **65** under atmospheric conditions which in turn underwent intramolecular Pictet–Spengler reaction through the addition of C-2 to the imine, to produce dihydropyridines **66**. Further oxidation converted **66** into pentacycles **67**. The same products were obtained when CoBr_2_ was used as external oxidant in the one-pot cascade reaction (Scheme 24, conditions b). No substantial improvement in the yields of **67** and **69** was observed when the synthesis was carried out in two steps, performing the GBB reaction under inert atmosphere and oxidizing **64** in the presence of CoBr_2_ (Scheme 24, conditions c and d; one-pot: 10–43%, two steps: 11–37% yields). Interestingly, polycycles **69** were formed when I_2_ was employed as the oxidant (Scheme 24, conditions e), which was presumably due to the coordination between the oxidant and the nitrogen atom in imidazoles **65**. This interaction increased the steric hindrance around C-2 and allowed the Pictet–Spengler reaction to occur at position of C-4. Indole-4-carbaldehydes were also compatible with this transformation, while indole-2-carbaldehyde (**70**) surprisingly led to the formation of linked polyheterocycles **75**. The authors then decided to perform the reaction using 3 equiv of aldehyde **70** under acidic conditions (Scheme 25, conditions a). The presence of a nucleophilic indole in the GBB adducts **71** induced further additions towards the excess aldehydes **70**. The diols **73** underwent cyclization and dehydration to produce linked polyheterocyclic indoles **75**. The authors managed to prepare five adducts in 15–33% yield. Another mechanistic scenario occurred at higher temperature (Scheme 25, conditions b). The secondary amine of diol **73** substituted both secondary alcohols to provide the ammonium **77**. The intermediate **77** underwent Steven rearrangement to give a bridged polyheterocycle **78** in 28% yield.

**Scheme 26:**
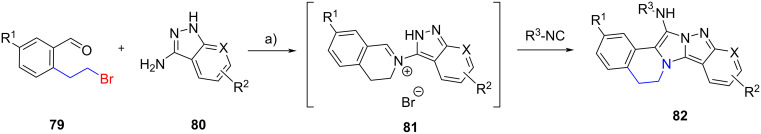
One-pot cascade synthesis of pentacyclic dihydroisoquinolines (X = N or CH). In blue the structural motif involved in the secondary transformation, in red the leaving group that is not incorporated in the final product. Conditions: a) *p*-TsOH (20 mol %), EtOH, 78 °C, 1 h.

Unlike the previous examples, Jeong et al. [60] developed a cascade reaction by installing the additional ring prior to the GBB reaction (Scheme 26). The bifunctional 2-(2-bromoethyl)benzaldehyde **79** was utilized to form the highly reactive cyclic iminium intermediates **81** through the condensation with amidines **80**. The subsequent formal [4 + 1] cycloaddition with the isocyanide and the 1,3-*H*-shift occurred to afford pentacycles **82**. Performing the one-pot reaction under the optimized conditions allowed them to create a library comprising of 26 polyheterocycles **82** with yields ranging from 71 to 94%. This protocol was scaled up to 10 mmol scale without losing the performance of the GBB multicomponent reaction.

**Scheme 27:**
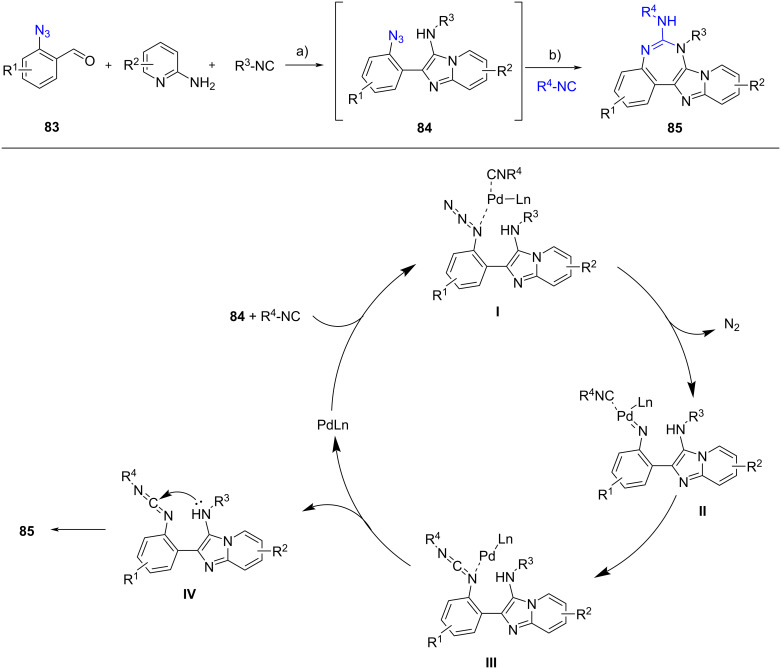
One-pot stepwise synthesis of imidazopyridine-fused benzodiazepines **85**. Conditions: a) *p*-TsOH (20 mol %), MeOH, rt, 24 h; b) Pd(OAc)_2_ (10 mol %), PhMe, 60 °C, 12 h.

Recently, Xiong et al. [61] developed a one-pot stepwise synthesis comprising a GBB-3CR and a palladium-catalyzed azide-isocyanide coupling to generate imidazo[1,2-*a*]pyridine-fused 1,3-benzodiazepines **85** (Scheme 27). The GBB reaction smoothly proceeded using 2-azidobenzaldehydes **83**, 2-aminopyridines and isocyanides as the precursors. The in situ-generated azides **84** were then reacted with a different isocyanide (R^4^–NC) in the presence of a palladium catalyst. The release of nitrogen from intermediates **I** resulted in nitrenes **II**, which in turn involved in the intramolecular transfer to yield species **III**. The carbodiimides **IV**, which were formed through reductive elimination of **III**, underwent intramolecular cyclization to deliver the desired products **85**. The scope of reaction showed that higher yield (57–90%) of **85** were obtained when benzaldehydes **83** were equipped with electron-donating groups (R^1^) and when bulky groups, such as 1-adamantyl or *t*-Bu (R^4^), were incorporated into the second isocyanides. The utility of this protocol was evaluated by conducting a gram-scale synthesis using 10 mmol of all precursors and there was no significant reduction in the yield of products.

**Scheme 28:**
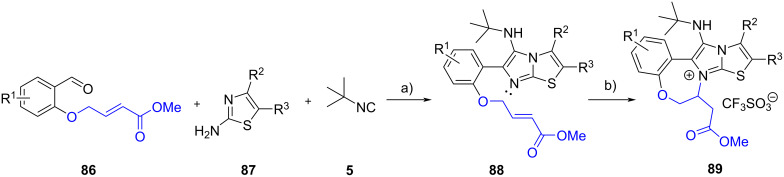
One-pot stepwise synthesis of benzoxazepinium-fused imidazothiazoles **89**. Conditions: a) Yb(OTf)_3_ (20 mol %), MeOH/DCM (3:1), rt, 12–15 h; b) Yb(OTf)_3_ (30 mol %), 70°C, 9–12 h.

Al-Tel et al. [62] developed a synthetic strategy where a Michael acceptor (a conjugated ester) was positioned in the aldehyde precursors **86** (Scheme 28). Various 2-aminothiazoles **87** and *tert-*butyl isocyanide (**5**) were reacted with **86** in the presence of Yb(OTf)_3_, affording **88**. Addition of a new aliquot of Yb(OTf)_3_ (30 mol %) allowed the new ring formation through a 7-*exo*-*trig* intramolecular aza-Michael addition, leading to the benzoxazepinium triflate salt **89**. To broaden the scope of the reaction, 2-aminopyrazine and 2-aminoquinoline were also introduced to the one-pot process, furnishing 6-7-5-6 and 6-7-5-6-6 polycycles, respectively (not shown).

**Scheme 29:**
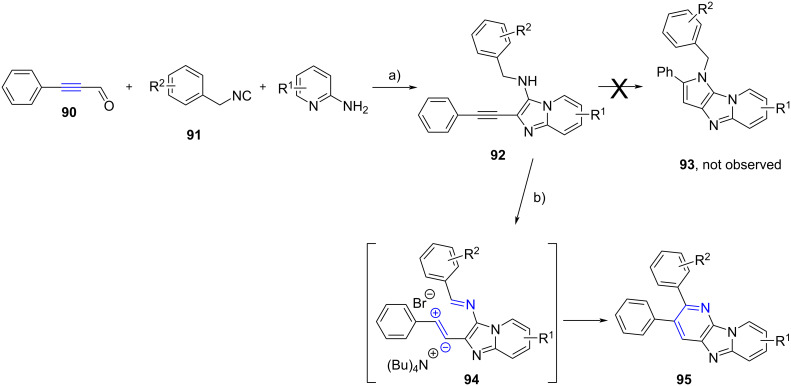
One-pot stepwise synthesis of fused imidazo[4,5,*b*]pyridines **95**. Conditions: a) HClO_4_, MeOH, rt, overnight; b) TBAB, DMF, 150 °C (MW), 30 min.

Chen et al. [63] developed an interesting strategy for the construction of new tricyclic systems (Scheme 29). Adducts **92** were prepared from 3-phenylpropiolaldehyde (**90**), benzylisocyanides **91** and 2-aminopyridines via a HClO_4_-promoted GBB reaction, then, under thermal conditions, in the presence of tetrabutylammonium bromide, an intramolecular nucleophilic addition of the secondary amines to the internal alkyne was expected to occur, forming a new pyrrole ring in **93** through a 5-*endo*-*dig* cyclization. Unexpectedly, they observed the generation of **95** with a new pyridine ring. The authors proposed that the benzylamine unit of **92** was oxidized to the corresponding imine **94** by oxygen in the air (as the cyclization did not proceed when the reaction was conducted under nitrogen atmosphere). The activation of the triple bond by tetrabutylammonium bromide regioselectively induced the 6-*endo*-*dig* cyclization to furnish **95** in moderate yields (41–67%).

**Scheme 30:**
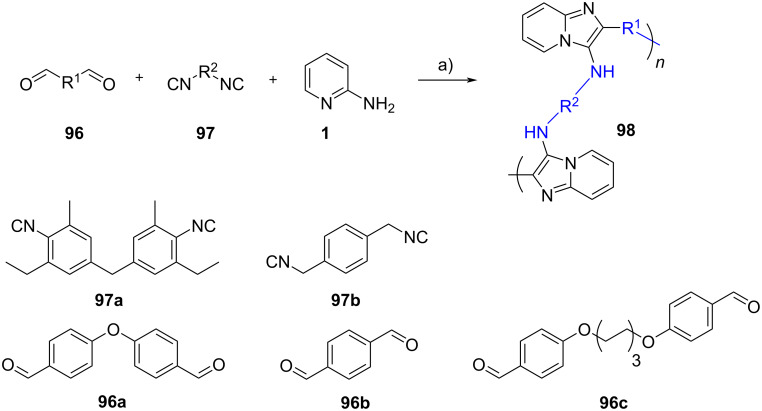
Synthesis of heterocyclic polymers via the GBB reaction. Conditions: a) *p*-TsOH, EtOH, 70 °C, 24 h.

Multicomponent polymerizations are simple and efficient methods to construct a library of polymers with great structural complexity and diversity, including fused-heterocyclic polymers[64]. Tang et al*.* [65] reported the transition-metal-free multicomponent polymerization of dialdehydes **96a–c** and diisocyanides **97a**,**b** in the presence of 2-aminopyridine (**1**, Scheme 30). The polymerization was carried out under mild conditions (in ethanol as the solvent and *p-*TsOH as the catalyst) and delivered six polymers featured with high yields (up to 98%), molecular weight (up to 41,700 g/mol), atom economy (water was the only byproduct) and thermal stability as well as remarkable fluorescence properties.

**Scheme 31:**
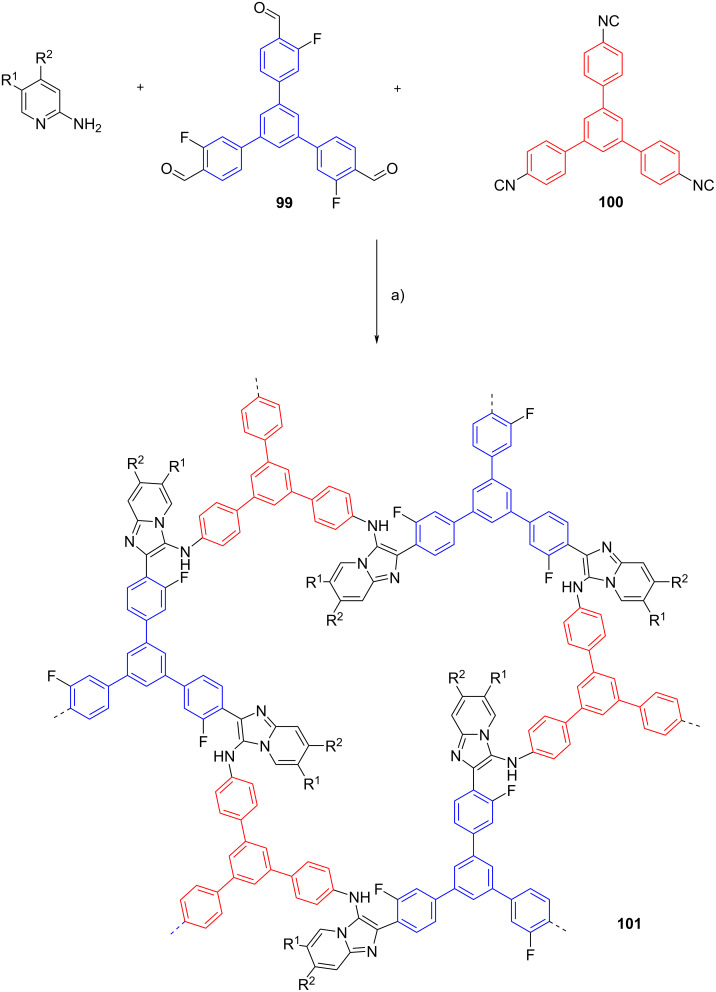
One-pot multicomponent reaction towards the synthesis of covalent organic frameworks via the GBB reaction. Conditions: a) *p*-TsOH, ethanol/mesitylene (1:3), 120 °C, 5 d.

Multicomponent reaction can be also employed to prepare crystalline porous materials namely covalent organic frameworks (COFs). The structural diversity and functionality of COFs were assembled from organic monomers via a different type of organic reaction [66]. Wang et al. reported for the first time the synthesis of COFs via the GBB reaction [67]. In their study, 1,3,5-tris(3-fluoro-4-formylphenyl)benzene (**99**), 1,3,5-tris(4-isocyanophenyl)benzene (**100**) and 2-aminopyridines were engaged in the GBB reaction, furnishing COFs **101** (4 examples in 70–77% yield) in one-pot fashion. *p*-TsOH was utilized as the catalyst and the multicomponent reaction was conducted in ethanol/mesytilene at 120 °C. However, the reaction took 5 days to complete (Scheme 31).

**Scheme 32:**
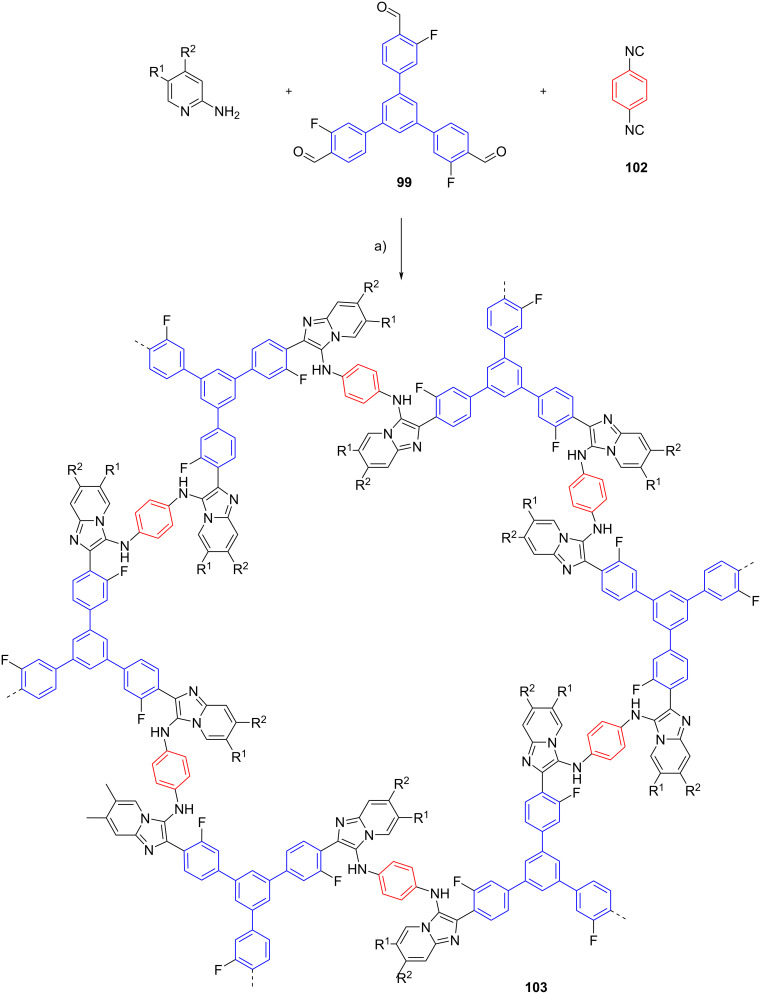
One-pot multicomponent reaction towards the synthesis of covalent organic frameworks via the GBB reaction. Conditions: a) *p*-TsOH, ethanol/mesitylene (1:3), 120 °C, 5 d.

The versatility of the GBB reaction in the synthesis of COFs was further demonstrated by employing the 1,4-diisocyanobenzene (**102**) monomer, leading to the generation of four COFs **103** in 71–88% yield. The results showed that the synthesized COFs displayed good chemical and thermal stability (Scheme 32).

#### 3.2 GBB-like reactions

**Scheme 33:**
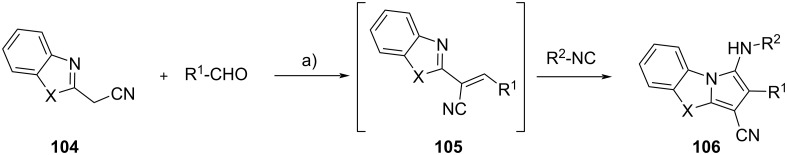
GBB-like multicomponent reaction towards the synthesis of benzothiazolpyrroles (X = S) and benzoxazolpyrroles (X = O). Conditions: a) 2-(hydroxymethyl)pyrrolidinium acetate salt (10 mol %), PhMe, 80–130 °C, 3 h.

Basak et al. [68] reported a variation of the GBB reaction employing benzothiazole acetonitrile **104** (X = S) in place of the amidine component (Scheme 33). Knoevenagel condensation with an aldehyde in the presence of 2-(hydroxymethyl)pyrrolidinium acetate produced conjugated imines **105** as the reaction intermediates. Once generated, the intermediates **105** and isocyanides underwent formal [4 + 1] cycloaddition followed by tautomerization, affording benzothiazolpyrroles **106** (X = S) in moderate to good yields (52–82%). In addition, performing the three-component reaction using benzoxazole acetonitrile **104** (X = O) or 2-pyridyl acetonitrile (not shown) led to the formation of benzoxazolpyrroles **106** (X = O) and indolizines (not shown), respectively.

**Scheme 34:**
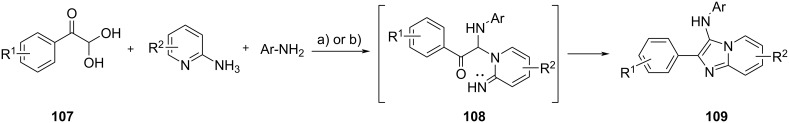
GBB-like multicomponent reaction towards the formation of imidazo[1,2,*a*]pyridines. Conditions: a) I_2_ (10 mol %), EtOH, 80 °C (MW), 45–300 min; or b) *p*-TsOH (20 mol %), EtOH, 55 °C, 1–1.5 h.

The GBB reaction is a powerful tool for the construction of imidazo[1,2,*a*]pyridines. Khan et al. [69] envisioned an isocyanide-free protocol based on arylglyoxals **107**, 2-aminopyridines and aromatic amines (Scheme 34). The GBB-like multicomponent reaction was carried out under MW heating. The initial condensation of the three precursors was catalyzed by I_2_, providing electron deficient ketoimines **108**. The intermediates **108** underwent intramolecular heterocyclization to furnish the fused products **109**, formally derived from a GBB reaction with aromatic isocyanides and benzaldehydes. The same approach was used by Jalani and Jeong [70] where *p*-TsOH served as the catalyst for the one-pot reaction. A library of 21 examples of **109** were produced in 41–78% yields. Various functional groups were found to be compatible to the reaction conditions and a gram-scale synthesis was carried out to demonstrate the versatility of this methodology.

#### 3.3 Post functionalization of GBB adducts

The structural complexity of the original scaffolds of the GBB adducts can be increased by decorating the structure of substrates (i.e*.*, aldehydes or isocyanides) with additional functional groups for further modifications. Unlike the one-pot synthesis strategy, the adducts were isolated before being subjected to the post functionalization process.

**Scheme 35:**
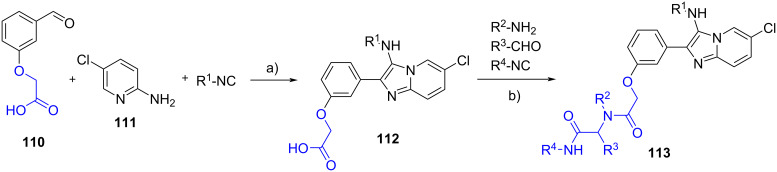
Post-functionalization of GBB products via Ugi reaction. Conditions a) HClO_4_, DMF, rt, 24 h; b) MeOH, 50 °C, 24–48 h.

Chebanov et al. [71] employed 2-(3-formylphenoxy)acetic acid (**110**), 2-amino-5-chloropyridine (**111**) and an isocyanide in a HClO_4_-promoted GBB reaction (Scheme 35). The use of a benzaldehyde bearing a carboxylic acid enabled heterocycles **112** to undergo a Ugi four-component reaction with additional aldehydes, isocyanides and primary amines; the corresponding Ugi adducts (20 examples) **113** were obtained in 28–72% yields.

**Scheme 36:**
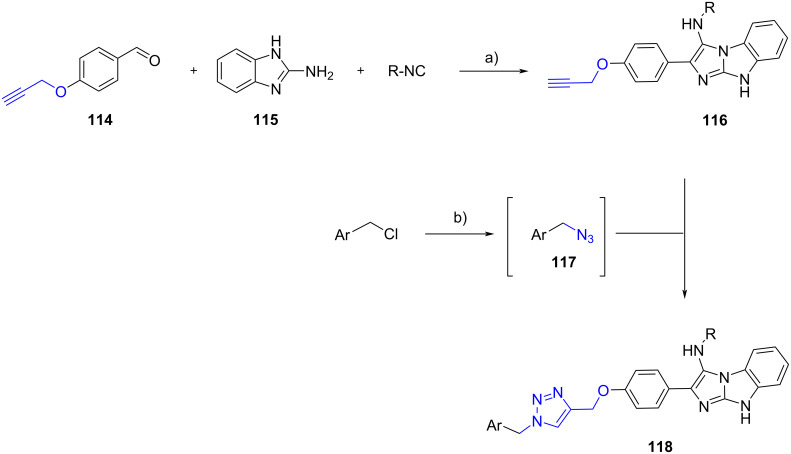
Post-functionalization of GBB products via Click reaction. Conditions: a) solvent-free, 150 °C, 24 h; b) NaN_3_, NEt_3_, CuSO_4_, sodium ascorbate, *t*-BuOH/H_2_O (1:1), rt, 24 h.

Mirza and Moghanlou [72] reported the solvent-free GBB reaction involving 4-(prop-2-yn-1-yloxy)benzaldehyde (**114**), 2-aminobenzimidazole (**115**) and isocyanides (Scheme 36). In this case, the additional alkyne group of **116** underwent a click [3 + 2] cycloaddition with the in situ-generated benzyl azides **117** to produce 15 examples of triazoles **118** in 63–88% yield.

**Scheme 37:**
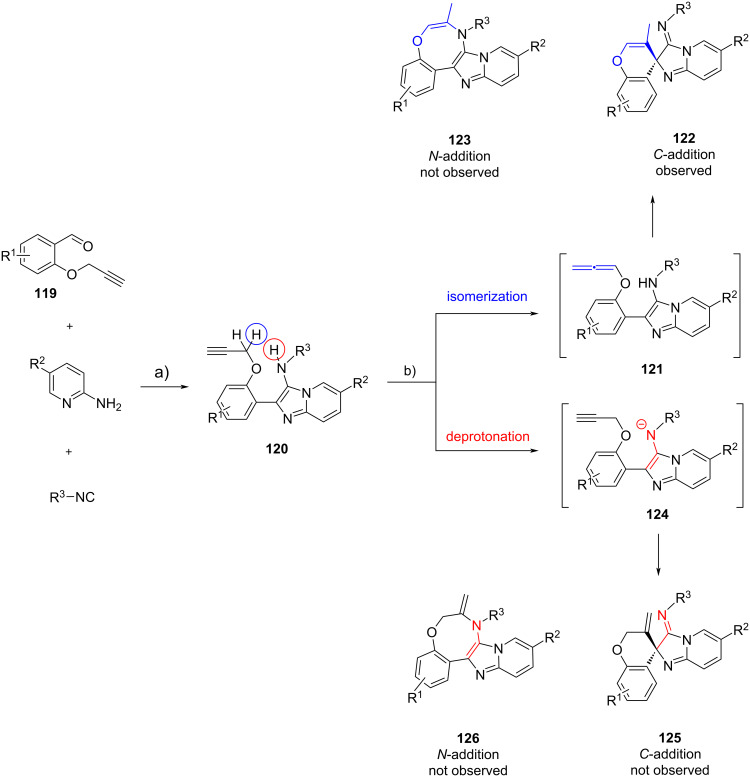
Post-functionalization of GBB products via cascade alkyne–allene isomerization–intramolecular nucleophilic addition. Conditions: a) InCl_3_ (10 mol %), MeOH, 55 °C, 12 h; b) KO*t*-Bu (4 equiv), DMSO, 55 °C, 4 h.

Balalaie et al. [73] demonstrated a fascinating chemo- and regioselective post-functionalization of GBB products **120** by exploiting the reactivity of the triple bond of 2-(propargyloxy)benzaldehydes **119** (Scheme 37). They discovered the formation of unexpected spiro[chromene-imidazo[1,2,*a*]pyridine]-3’-imines **122** when the GBB precursors **120** were treated under basic conditions using 4 equivalents of KO*t*-Bu. They managed to create a small library of 15 spirocycles **122** in moderate to high yields (53–89% yields). The mechanism of spirocyclization reaction was studied using density functional theory (DFT). The DFT studies revealed that there were two possible mechanistic pathways. In the first scenario, the deprotonation of the propargyloxy group of **120** would trigger the alkyne–allene isomerization and the allene intermediates **121** were formed. The further treatment of allenes **121** using the excess KO*t*-Bu would induce the subsequent intramolecular nucleophilic *C*-addition (by imidazole carbon) or *N*-addition (by amine nitrogen) to allenes, leading to the generation of spirocycles **122** (via 6-*exo*-*dig* cyclization) or fused heterocycles **123** (via 8-*exo*-*dig* cyclization), respectively. In the second scenario, the treatment of the GBB products **120** using KO*t*-Bu could lead to the deprotonation of the secondary amine, generating nitrogen anionic intermediates **124**. Then, the spiro **125** and fused **126** products could be generated via the intramolecular nucleophilic *C*-addition or *N*-addition to the terminal alkynes. The DFT studies showed that the propargyloxy group displayed more thermodynamic acidity, whereas the amine showed more kinetic acidity. The potential energy surface can explain the selectivity of the reaction where the intramolecular nucleophilic addition was the rate-determining step in favor to the formation of spiro products **122** relatively to **123**, **125**, and **126**. Based on the calculation, the addition to allenes was more favorable than that to alkynes, as well as intramolecular nucleophilic *C*-addition compared to *N*-addition.

**Scheme 38:**
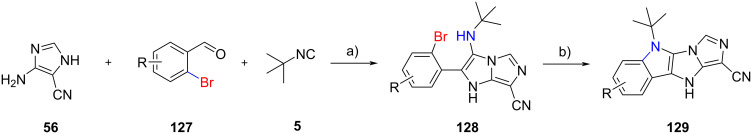
Post-functionalization of GBB products via metal-catalyzed intramolecular *N*-arylation. In red and blue the functional groups involved in the Buchwald N-arylation. Conditions: a) HClO_4_, MeOH, rt, 24 h; b) Pd(OAc)_2_ (10 mol %), Xantphos, K_3_PO_4_, 1,4-dioxane, 120 °C, 2 h.

The combination of a GBB reaction with a Buchwald *N*-arylation was already reported by Guillamet et al. in 2012 [74], employing aminopyridines and aromatic aldehydes bearing a bromine atom in the *ortho*-position. In 2019, the same authors have reported an extension of the previous methodology using 5-amino-4-cyano-1*H*-imidazole (**56**) as amidine component (as described in the previous chapter), generating this time the imidazo[5′,1′:2,3]imidazo[4,5-*b*]indole scaffold **129** from adduct **128** (Scheme 38) [55]. The Buchwald reaction was found to be tolerant to both electron-donating (R = Me, 72% yield) and electron-withdrawing (R = F, 61% yield) groups displayed by aldehydes **127**, however, bromoheteroaryl aldehydes were unsuccessful.

**Scheme 39:**
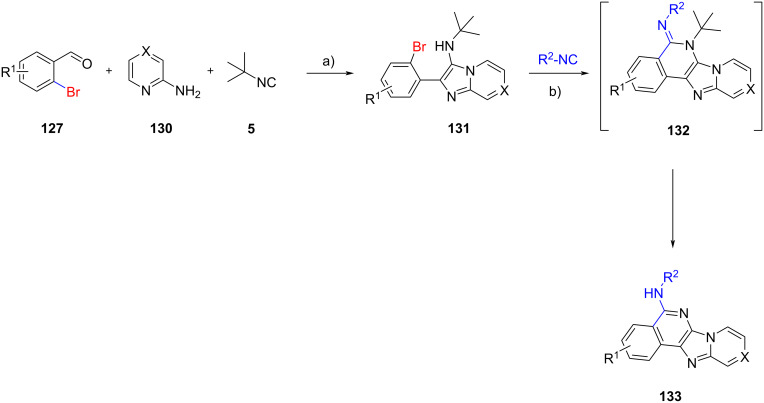
Post-functionalization of GBB products via isocyanide insertion (X = N or CH). Conditions: a) HClO_4_, MeOH, rt, 3 d; b) Pd-Peppsi, XPhos, KOAc, DMF, 120 °C, 24 h.

The intramolecular *N*-arylation strategy could be extended through isocyanide insertion as demonstrated by Bräse et al. [75]. The authors proposed that the insertion of a second molecule of isocyanide on the GBB adduct **131**, derived from bromoaldehydes **127**, 2-aminopyridines **130** and *tert*-butyl isocyanide (**5**), could take place between the secondary amine and the bromide, affording the imine intermediate **132** in the presence of a mild base like potassium acetate. The elimination of the *tert*-butyl group as isobutene gas, followed by tautomerization and aromatization, led to the generation of imidazo[4,5-*c*]isoquinolines **133** (Scheme 39). The use of a stronger base in the Buchwald reaction was not carried out since the *N*-arylation would be accelerated (compared to isocyanide insertion), yielding the corresponding five-membered ring as in Scheme 38. A small set of 13 polyheterocycles **133** was prepared with yields ranging from 20 to 94%.

**Scheme 40:**
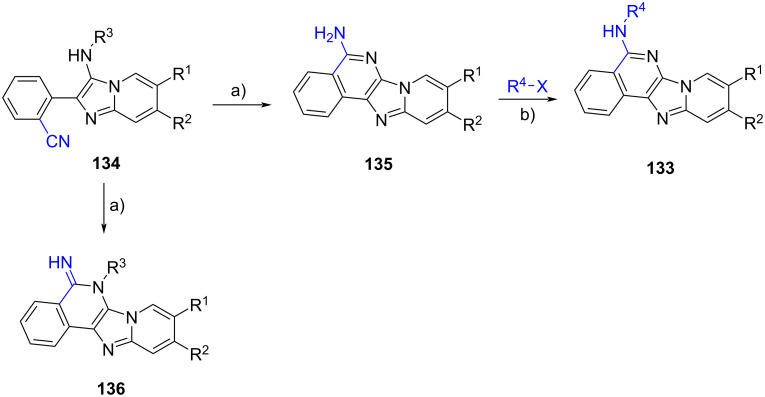
Post-functionalization of GBB products via intramolecular nucleophilic addition to nitriles. Conditions: a) TFA/DCM (1:1), rt; b) Pd(OAc)_2_, Xantphos, Cs_2_CO_3_, PhMe, 120 °C.

Another strategy to access polyheterocyclic motif **133** was employed by Berteina-Raboin et al. [76] (Scheme 40). A series of GBB adducts **134** were initially prepared from 2-formylbenzonitriles, 2-aminopyridines and isocyanides under the reaction conditions optimized in their previous publication [77]. When R^3^ = *t*-Bu, the treatment of the multicomponent products **134** with TFA/DCM (1:1) facilitated the removal of the *t*-Bu group and the activation of the nitrile, leading to the formation of ten primary amines **135** with yields ranging from 65 to 92%. The primary amines **135** were then engaged with various aryl halides via Buchwald–Hartwig cross coupling reaction to generate the substituted polyheterocycles **133**. When cyclohexyl- and benzyl-tagged amines **134** (R^3^ = Cy and Bn) were subjected to the intramolecular cyclization under acidic conditions, imines **136** were obtained in 80% and 78% yields, respectively.

**Scheme 41:**
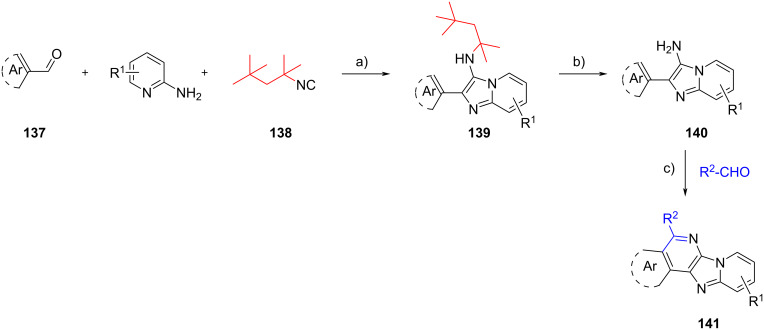
Post-functionalization of GBB products via Pictet–Spengler cyclization. Conditions: a) 4 N HCl/dioxane, MeCN, 110 °C (MW), 20 min; b) TFA/DCM (1:1), rt, 25 min; c) *p*-TsOH, PhNO_2_, 160 °C, 6 h.

The isocyanide-derived amino group of the GBB adducts can be involved also in a Pictet–Spengler reaction, as reported by Salunke et al. (Scheme 41) [78]. In this study, electron-rich aromatic aldehydes **137** were used as GBB substrates together with 2-aminopyridines and 1,1,3,3-tetramethylbutyl isocyanide (**138**). Upon completion of the multicomponent condensation, the *tert*-octyl group of **139** was removed by TFA, liberating primary amines **140**. *p*-TsOH-catalyzed Pictet–Spengler cyclization of primary amines **140** with other aromatic/aliphatic aldehydes (R^2^-CHO) gave fused imidazo[4,5,*b*]pyridine derivatives **141**. By employing this protocol, the authors managed to produce 15 examples of polycyclic heterocycles **141** in 30–87% yields. The study of the reaction scope showed that the electronic effect on aromatic aldehydes (R^2^–CHO) did not affect the cyclization reaction, however, lower yields were observed when aliphatic aldehydes were used as precursors, due to the instability of the Schiff base generated during the reaction.

**Scheme 42:**
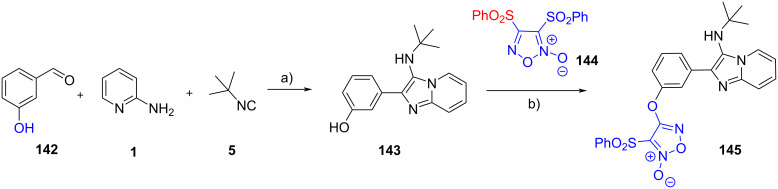
Post-functionalization of GBB products via *O*-alkylation. Conditions: a) TFA (20 mol %), EtOH, 120 °C (MW), 20 min; b) NaOH, 2-MeTHF, rt. 24 h.

In 2022, López et al. [79] prepared the hybrid compounds **145** by incorporating the furoxan moiety into the GBB adduct (Scheme 42). The multicomponent reaction was initially performed from 3-hydroxybenzaldehyde (**142**), 2-aminopyridine (**1**) and *tert*-butyl isocyanide (**5**). In the second reaction, **143** was then coupled with 3,4-bis(phenylsulfonyl)furoxan (**144**) under basic conditions to furnish GBB-furoxan hybrid **145**, albeit in low yield (23%).

**Scheme 43:**
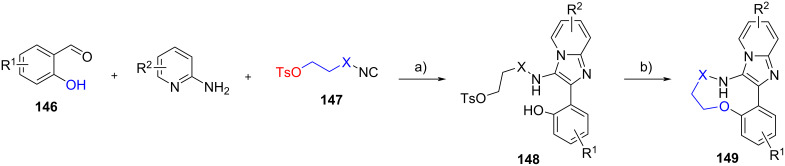
Post-functionalization of GBB products via macrocyclization (X = -CH_2_CH_2_O-, -CH_2_-, -(CH_2_)_4_-). Conditions: a) Sc(OTf)_3_ (20 mol %), MeOH, rt, 24 h; b) DBU, DMF, 80 °C, 15–18 h.

Finally, Neochoritis et al. [80] exploited an intramolecular aliphatic nucleophilic substitution to obtain macrocycles. In this study, isocyanides were equipped with a tosylate leaving group. A range of substrates **147**, with different length and motif of the tethering groups, were prepared from the corresponding amino alcohols. Bifunctional derivatives **147** were then reacted with 2-hydroxybenzaldehydes **146** and 2-aminopyridines, affording adducts **148** in quantitative yields (Scheme 43). The subsequent treatment of **148** with DBU allowed the formation of 9-, 11-, and 13-membered macrocycles **149** with yields up to 69% (9 examples). The authors also demonstrated that 3-hydroxybenzaldehyde could be employed as the substrate for this transformation. Besides the GBB reaction, other multicomponent reactions such as Ugi–Smiles, classical Ugi four-component and tetrazole variation of Ugi reactions could be combined with the macrocyclization step to deliver numerous novel macrocyclic scaffolds.

### 4 Novel biological activities

Many GBB adducts displayed a variety of biological activities. In recent years, the researchers have evaluated their pharmacological properties by performing in vitro assays, such as antimicrobial (against bacteria and virus), anticancer (inhibition against cancer cell lines, tumor cells and proteins responsible for mechanisms of action) and anti-inflammatory. In some reports, the in silico studies (through molecular docking), ADMET predictions and structure–activity relationships were also carried out to evaluate the therapeutic potentials of the GBB products in drug development.

#### 4.1 Antimicrobial activity

**Figure 1:**
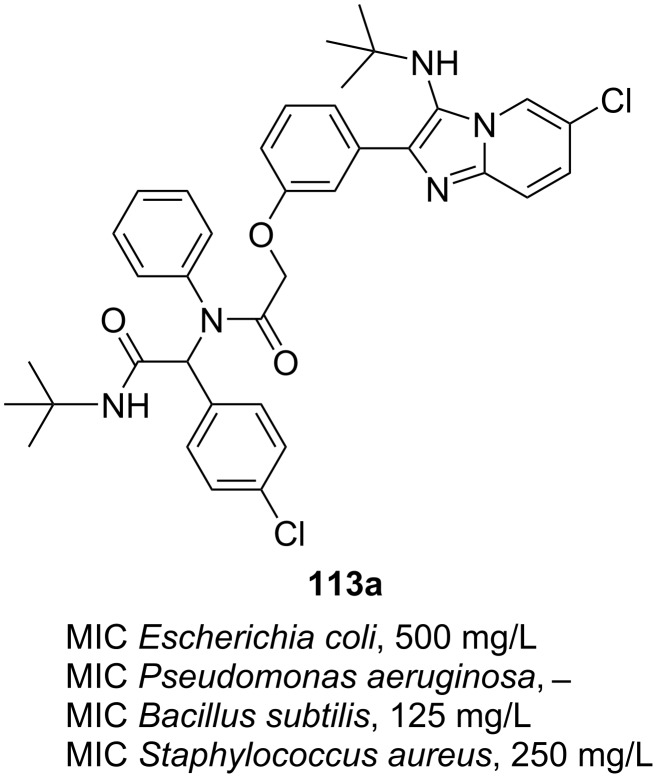
Antibacterial activity of GBB-Ugi adducts **113** on both Gram-negative and Gram-positive strains.

In 2023, Chebanov et al. [71] tested 9 compounds **113**, derived from the combination of GBB and Ugi type reactions (see Scheme 35), as antibacterial agents. There were four bacteria strains used in this study including Gram-negative of *Escherichia coli* strain 1257 and *Pseudomonas aeruginosa* strain 1111 as well as Gram-positive of *Bacillus subtilis* strain 1211 and *Staphylococcus aureus* strain 2231. Nitroxoline was employed as positive standard. Some of the synthesized adducts showed weak inhibition to the growth of four bacteria strains. Product **113a** (Figure 1) displayed bacteriostatic activity against *B. subtilis* at a concentration of 125 mg/L. It inhibited the growth of *S. aureus* and *E. coli* with MICs of 250 and 500 mg/L, respectively. The strain of *P. aeruginosa* was found to be resistant to most of adducts **113**.

**Scheme 44:**
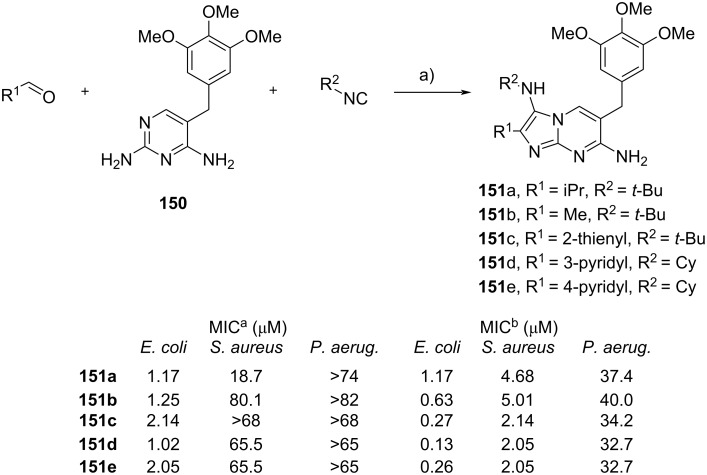
GBB multicomponent reaction using trimethoprim as the precursor. Conditions: a) Yb(OTf)_3_ or Y(OTf)_3_ (20 mol %), MeCN, 80 °C. The table reports the antibacterial activity of the synthesized compounds against various bacteria strains. ^a^Minimum inhibitory concentration (MIC, μM) of the GBB products; ^b^minimum inhibitory concentration (MIC, μM) of the GBB products in combination with sulfamethoxazole (1:20).

Molecular modification is an important strategy in drug discovery and development, in which the structure of known drug molecules is chemically altered to improve their physico-chemical and pharmacodynamic/pharmacokinetic properties [81–82]. Lavilla et al. [83] developed antimicrobial agents by modifying the structure of trimethoprim (TMP, **150**), an antibiotic used to treat diseases due to bacterial infection such as bladder or middle ear infections and acute diarrhea. By taking advantage of the 2-aminopyrimidine group, TMP (**150**) along with various aldehydes and isocyanides were introduced into the multicomponent reaction to generate the GBB adducts **151** with yields ranging from 6 to 59% (Scheme 44). Then, 15 compounds were evaluated for their antibacterial activity against *Escherichia coli* ATCC 25922, *Pseudomonas aeruginosa* PAO1 and *Staphylococcus aureus* ATCC 29213. The biological assay demonstrated that several GBB adducts (**151a**–**e**) were as potent as the marketed antibiotic TMP, where all tested compounds were more active against *E. coli* than *S. aureus*. The results indicated that the molecular modification strategy did not affect the synthesized compounds to penetrate the outer membrane of Gram-negative bacteria. However, *P. aeruginosa* was resistant to all GBB adducts and TMP standard. In addition, the synergistic effect was observed when all compounds **151a**–**e** were combined with sulfamethoxazole (1:20) in the in vitro assay against *E. coli* and *S. aureus*. The latter was found to be more sensitive to the drug combination than the individual compound. The drug synergism of all adducts **151a**–**e** with sulfamethoxazole was also effective to inhibit several clinical methicillin-resistant *Staphylococcus aureus* (MRSA) isolates, such as *S. aureus* 8125304770, 8139265926, 8125255044 and 8124825998 (not shown). Unfortunately, the drug combination with sulfamethoxazole showed no effect against *P. aeruginosa.*

**Figure 2:**
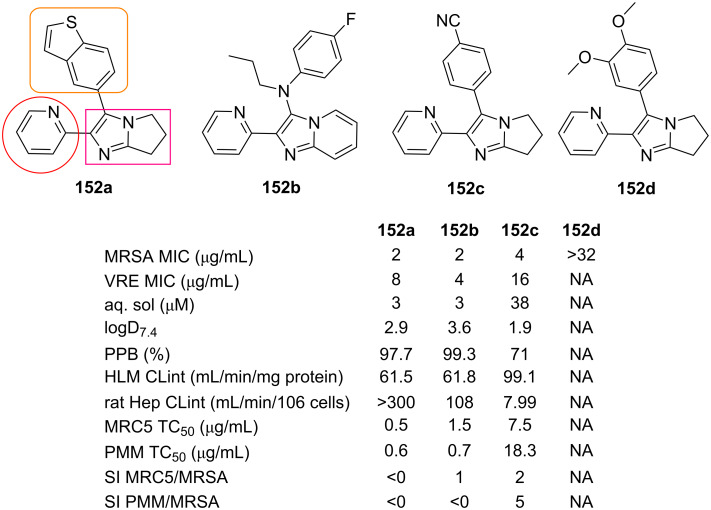
Antibacterial activity of GBB adducts **152** against MRSA and VRE; NA = not available.

Todd et al. [84] investigated the potential of 147 heterocycles containing fused imidazole scaffolds (where some of them (i.e., **152b**) were prepared through GBB multicomponent reaction) as antibacterials against methicillin-resistant *Staphylococcus aureus* (MRSA). A structure–activity relationship (SAR) study was carried out through the diversification of a bicyclic imidazole moiety with two (hetero)aryl substituents (in Figure 2 representative examples **152a**–**d** are reported). The in vitro assay revealed that 43 examples (including **152a**, **152b**, **152c**) showed promising antibacterial potency against MRSA with MIC ≤ 8 μg/mL. While 11 examples displayed moderate activity (MIC = 16 μg/mL), 88 examples were inactive (MIC > 32 μg/mL). Some products were also tested against vancomycin-resistant enterococcus (VRE), where **152b** displayed a low MIC value of 4 μg/mL.

The ADME prediction was then performed to evaluate the pharmacodynamics and pharmacokinetic properties of **152**, including thermodynamic aqueous solubility (≥10 μM), logD_7.4_ value (<2), human PPB (≤95%), human liver intrinsic clearance (HLM CL_int_ < 9.0 mL/min/mg protein), and rat hepatocyte intrinsic clearance (rat hep CL_int_ < 5 mL/min/106 cells). The toxicity of the tested compounds was also carried out against the human lung fibroblast cell line MRC-5_SV2_ and primary mouse macrophages (PMM). The selectivity indices (SI, ratio of TC_50_ to MRSA MIC) were then calculated to evaluate the mammalian toxicity of the fused imidazole derivatives. Evaluation of MIC values, physicochemical properties and selectivity indices showed that compound **152c** could be a promising antibacterial agent against MRSA.

A kinase target selectivity study was then applied to HEK293 cell lysates to comprehend the mechanism of action. In this case, the active (**152a**) and inactive (**152d**) compounds were used as the representative examples. The results demonstrated that the potential mammalian target for the tested compounds could be TGF-β1.

**Figure 3:**
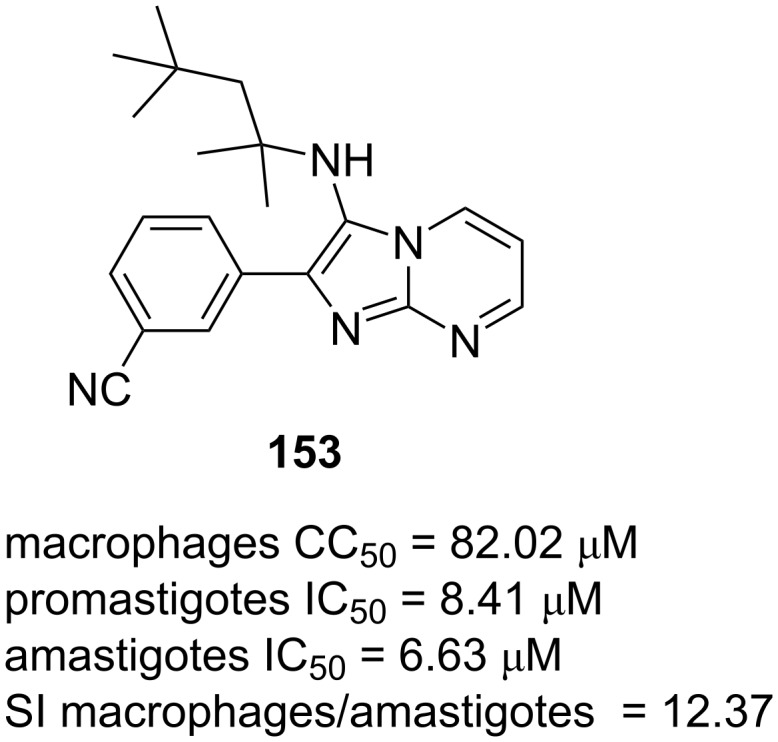
Antibacterial activity of GBB adduct **153** against *Leishmania amazonensis* promastigotes and amastigotes, determined through growth inhibition assays.

Salunke et al. [85] have created a library of 28 imidazo[1,2-*a*]pyrimidines and evaluated their in vitro antileishmanial activity against *Leishmania amazonensis* promastigotes and amastigotes. Among them, the GBB adduct **153** (Figure 3) displayed significant inhibition to the growth of promastigotes (IC_50_ = 8.41 μM) and intracellular amastigotes (IC_50_ = 6.63 μM). The authors found that the presence of a *tert*-octyl group at the secondary amine unit was important for biological activity. Compound **153** exhibited better activity than the reference drug miltefosine and showed low toxicity to the macrophages host cell (CC_50_ = 82.02 μM and SI macrophages/amastigotes = 12.37) as well as human-origin cancer cell lines of MCF-7, MDA-MB-231 and HEP-G2.

The in silico study showed that compound **153** followed the Lipinski’s rule of five, indicating that it displayed excellent bioavailability. Based on ADMET prediction, it is an inhibitor of several CYP450 enzymes and could be used through topical or oral administration.

ATP synthase is an essential enzyme for the growth of *Mycobacterium tuberculosis* and has been considered as potential target for antitubercular drugs [86]. Jain and Sen [58] investigated the potential of indole-substituted-imidazo[1,2-*a*]pyridines **61** (Scheme 23) as antitubercular agents. A series of 14 indole-substituted-imidazo[1,2-*a*]pyridines **61** were subjected to the ATP synthase inhibition assay. All compounds agreed the parameters of the Lipinski’s rule of five. However, they showed a low level inhibition of ATP synthase.

**Figure 4:**
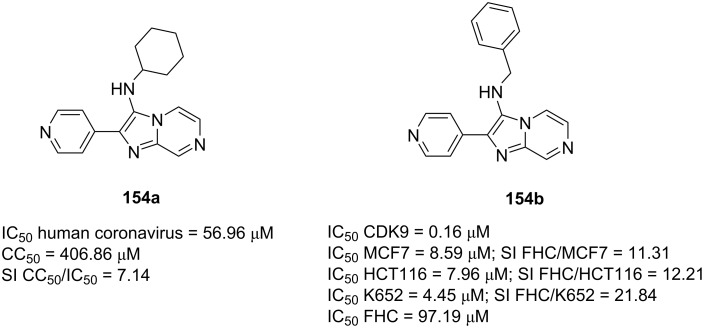
Antiviral and anticancer evaluation of the GBB adducts **154a** and **154b**. In vitro antiproliferative activity was determined against several cell lines using the MTT assay.

Alsfouk et al*.* [87] have prepared fused imidazo[1,2-*a*]pyrazines **154** via GBB multicomponent reaction and evaluated their potential antiviral and anticancer activities (Figure 4). Among 4 tested compounds, adduct **154a** exhibited good antiviral activity against human coronavirus 229E with the IC_50_ value of 56.96 μM and no cytotoxicity on the target cells at CC_50_ of 406.86 μM. Both antiviral activity and selectivity indices of **154a** were even better than those of the standard drug ribavirin. Compound **154a** was then docked into SARS-Cov-2 main protease. The molecular docking studies demonstrated the key hydrogen bond interactions between Cys44 and the *N*-pyridine unit of **154a** with a binding free energy of −7.6 kcal/mol.

#### 4.2 Anticancer activity

Imidazo[1,2-*a*]pyrazines **154** described above [87] have been tested by the same authors against cyclin-dependent kinase-9 (CDK9), a molecular target for the treatment of various malignant cancers, responsible for the cell cycle control and the promotion of cancer initiation as well as progression [88]. Derivative **154b** (Figure 4) exhibited promising inhibition potency on CDK9 with an IC_50_ of 0.16 μM. The in vitro antiproliferative activity of **154** was determined using the MTT (3-[4,5-dimethylthiazol-2-yl]-2,5 diphenyltetrazolium bromide) assay against several cancer cell lines including breast cancer (MCF7), colorectal myelogenous leukemia (K652) and colorectal cancer (HCT116). It was found that there was a correlation between cytotoxicity of **154** and their inhibitory activity against CDK9, indicating that the inhibition of CDK9 was the mechanism of action. Derivative **154b** was the most potent compound and superior to standard staurosporine. Moreover, it was more selective on cancer cell lines than on normal non-cancerous FHC cells. Molecular docking studies were performed into the ATP binding site of CDK9 and revealed a key interaction with Ile25 with a docking score of −8.3 kcal/mol. Based on in silico prediction, **154b** showed good drug-likeliness and displayed good oral bioavailability.

**Figure 5:**
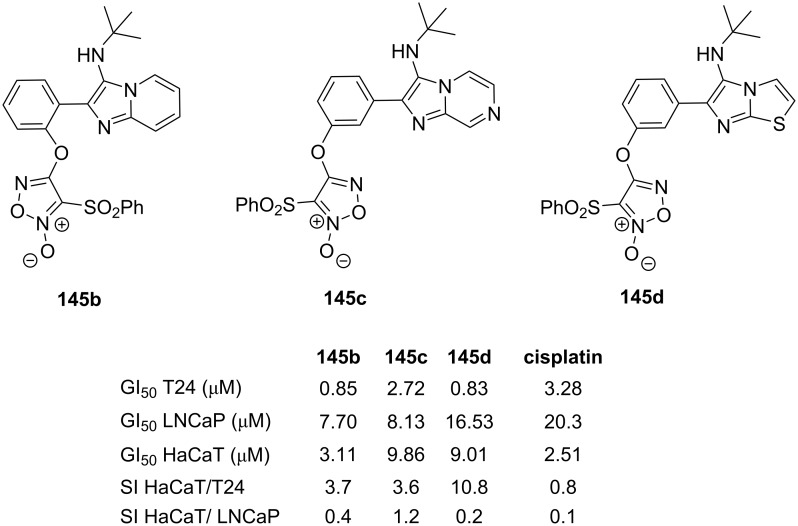
Anticancer activity of the GBB-furoxan hybrids **145b**, **145c** and **145d** determined through antiproliferative assays on various cell lines.

The anticancer activity against bladder cancer (T24) and prostate cancer (LNCaP) of previously described (Scheme 42) GBB-furoxan hybrids **145** was carried out by López et al. [79] by using the sulforhodamine B method. The results (Figure 5) demonstrated that hybrids **145b**, **145c**, and **145d** were more cytotoxic towards T24 and LNCaP cell lines than the reference compound cisplatin (GI_50_ T24 = 3.28 μM and GI_50_ LNCaP = 20.30 μM). Precursor **143**, lacking the furoxan scaffold (Scheme 42) showed no activity (GI_50_ > 100 μM) on both T24 and LNCaP cell lines, demonstrating the importance of the furoxan group. The antiproliferative activity of **145** against non-cancer cells (HaCaT cells, human keratinocyte) was then performed to determine the cytotoxic selectivity (SI = GI_50_ HaCaT/GI_50_ cell lines). While all compounds were selective toward T24 cancerous cells, showing better selectivity than the reference compound cisplatin, no selectivity was observed concerning the LNCaP cell line.

To elaborate the possible anticancer mechanisms of GBB-furoxan hybrids **145**, the authors carried out a nitric oxide (NO) release assessment [89]. The results showed that various levels of NO were produced in both T24 and LNCaP cancer cell lines by most of the compounds, however, there was no direct correlation between the antiproliferative activity and the amount of NO released.

**Scheme 45:**
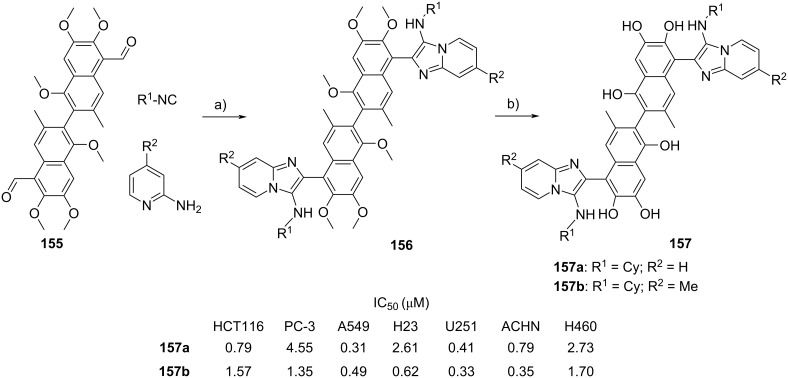
Synthesis and anticancer activity of the GBB-gossypol conjugates. Conditions: a) Sc(OTf)_3_ (10 mol %), DCM/MeOH (2:1), rt; b) BBr_3_ (25 equiv), DCM, rt, then diluted HCl. Antiproliferative assays were carried out against 7 different cell lines.

Li et al. have synthetized 13 GBB-gossypol conjugates and evaluated them as anticancer agents [90]. Methylated gossypol **155**, 2-aminopyridines and isocyanides were transformed into double GBB adducts **156**, which in turn underwent demethylation to **157** with yields ranging from 78 to 94% yields (Scheme 45).

The antiproliferative assay on **157** was carried out against 7 cancer cell lines including HCT116, PC-3, A549, H23, U251, ACHN and H460. According to their previous study [91], hydrophobic groups embedded into the gossypol structure improved the antiproliferative activity: GBB conjugates **157a** and **157b**, bearing a cyclohexyl group, were the most potent, compared to the conjugates bearing other alkyl or aromatic hydrophobic groups. The average IC_50_ on 7 cancer cells of **157a** was 1.74 μM. The cancer inhibition was enhanced when electron-donating groups were installed on the imidazopyridine unit (**157b**, R^2^ = Me, average IC_50_ value of 0.91 μM). The mechanism of action, based on molecular modelling analyses and binding assays, demonstrated a possible interaction with antiapoptotic proteins of the Bcl-2 family.

**Figure 6:**
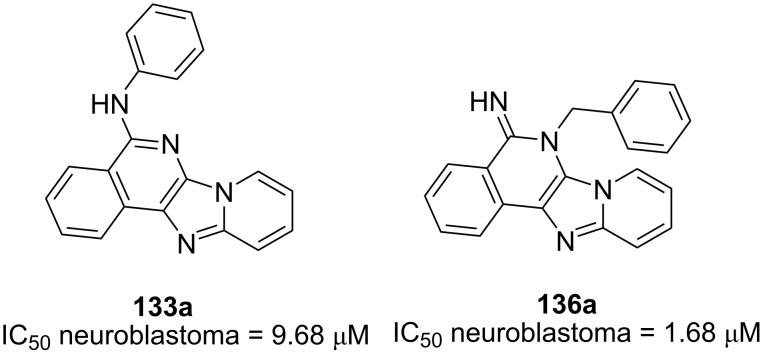
Anticancer activity of polyheterocycles **133a** and **136a** against human neuroblastoma. Clonogenic assays were carried out to determine the antiproliferative activity.

Previously described pyrido[2’,1’:2,3]imidazo[4,5-*c*]isoquinolin-5-amines **133** and pyrido[2',1':2,3]imidazo[4,5-*c*]isoquinolin-5(6*H*)-imines **136** (Scheme 40) have been tested for their anticancer activity against pediatric solid tumor neuroblastoma (SH-SY5Y cells) [76]. Derivatives **133a** and **136a** exhibited antiproliferative activity below 10 μM in clonogenic assays (Figure 6). The apoptotic effects of **133a** and **136a** were validated through the upregulation of apoptotic proteins of cleaved caspase-3, cleaved PARP-1 and Bax.

The molecular docking analysis was conducted using **133a** and **136a** as ligands and pro-apoptotic Bax protein as the target. The protein active site was positioned around the α1 and α2 helices. Compounds **133a** and **136a** showed docking scores of −6.7 and −6.9 kcal/mol, respectively. Both ligands generated important interactions with amino acid residues of Pro49, Pro51 and Leu25. Derivative **136a** also exhibited additional π-donor H-bond interactions with Gln52 and Asp53. The modelling study also showed that the interaction with the ligands could trigger the transformation of the α1–α2 loop from the closed conformation to the open one and this change of conformation could initiate the Bax activation, leading to the death of neuroblastoma cells.

**Figure 7:**
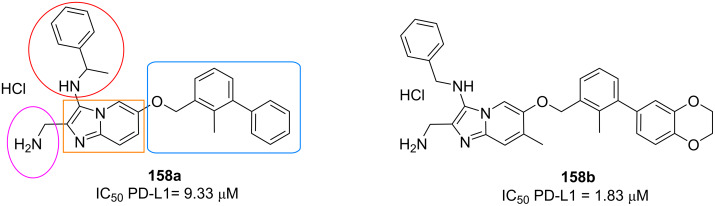
Development of GBB-adducts **158a** and **158b** as PD-L1 antagonists. HTRF assays were carried out against PD-1 and PD-L1 proteins.

Programmed death-1 (PD-1) is a co-inhibitory receptor which suppresses the activity of T-cells through the regulation of the TCR signaling. High expression of programmed death ligand-1 (PD-L1) in the microenvironment of tumors is frequently detected in various types of cancer. The binding of PD-L1 to PD-1 leads to T-cell disfunction, therefore blockage of the PD-L1/PD-1 interactions is a promising strategy in the development of anticancer agents [92–93]. Dömling et al. have designed, synthesized and evaluated 11 imidazo[1,2-*a*]pyridines **158** as PD-1/PD-L1 antagonists [94]. The design was conducted by incorporating a biphenyl, a methanamine and an amino group into the bicyclic heterocycle. The homogeneous time-resolved fluorescence (HTRF) assay against PD-1 and PD-L1 proteins showed that the compounds were able to disrupt PD-L1/PD-1 interactions. The presence of a dioxane moiety on the biphenyl unit and of a methyl group on the bicyclic ring lowered the IC_50_ values. The compounds with most promising PD-L1 inhibitory activity were **158a** and **158b** (Figure 7).

The use of topoisomerase (TOP1) poisons can kill cancer cells through the trapping of TOP1 on DNA, leading to lethal DNA double-strand breaks. A mechanism employed by cancer cells to resist killing by TOP1 poisons is the overexpression of enzymes capable to repair TOP1-DNA breaks, such as tyrosyl-DNA-phosphodiesterase 1 (TDP1). Therefore, the combination of TDP1 inhibitors and TOP1 poisons could synergistically be more effective for the treatment of cancer [95–96].

**Figure 8:**
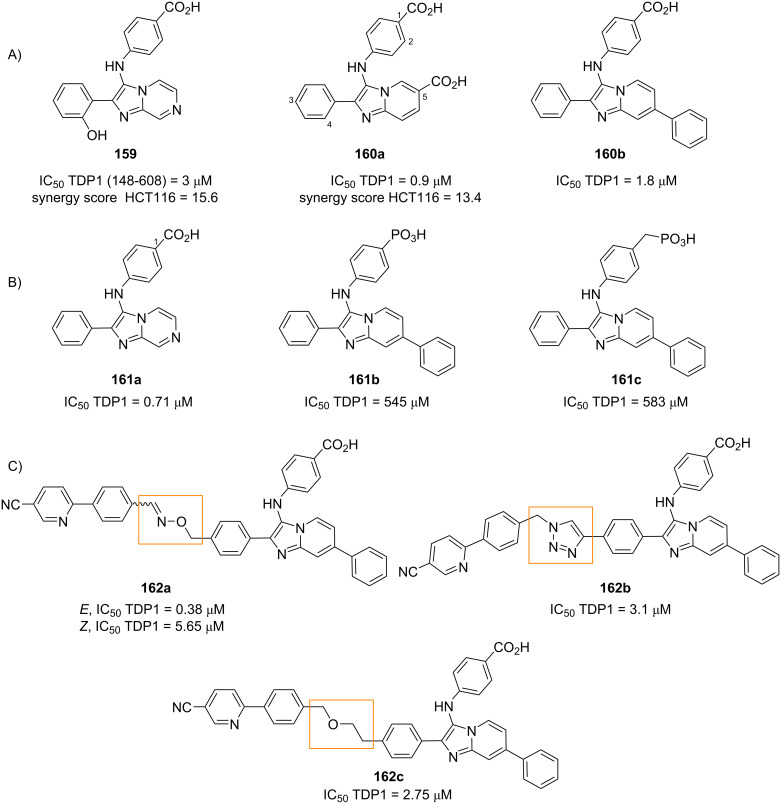
Development of imidazo[1,2-*a*]pyridines and imidazo[1,2-*a*]pyrazines as TDP1 inhibitors. The SMM method was used to identify new leads possessing TDP1 inhibition, determined through in vitro gel-based fluorescence assays.

Burke, Jr. et al. have developed various imidazo[1,2-*a*]pyridine- and imidazo[1,2-*a*]pyrazine-based TDP1 inhibitors. In a first study [97] they exploited the small molecule microarray (SMM) method to identify new leads possessing TDP1 inhibition; a library of 21,000 compounds was screened generating 37 lead compounds, and among them imidazo[1,2-*a*]pyrazine derivative **159** exhibited micromolar TDP1 inhibition (Figure 8A).

Based on these findings, the authors synthesized a set of 23 imidazo[1,2-*a*]pyridines and imidazo[1,2-*a*]pyrazines via GBB reaction and evaluated their TDP1 inhibitory potency. The structure–activity relationship demonstrated that the presence of carboxylic groups at positions 1, 2 and 5 (Figure 8A) was important for TDP1 inhibition, while a carboxylic acid at position 4, as well as proton donors (such as CO_2_H SO_3_H and OH) at position 3, resulted in loss of inhibitory activity. On the other hand, introducing non-proton donor groups (such as CH_3_, OBn, CF_3_ and NO_2_) at position 3 was found beneficial. Moreover, the authors demonstrated that their TDP1 inhibitors synergistically acted with TOP1 poison camptotechin (CPT) in human colon cancer (HCT116) cell line, with synergy scores larger than 10.

As a continuation of their research, Burke, Jr. et al. designed phosphonic acid-containing variants of their previously reported inhibitors. X-ray crystal structures of phenylphosphonic acid- and benzylphosphonic acid-containing compounds **161b** and **162c** (Figure 8B) revealed that the phosphonic acid headgroup had different binding modes compared to the bioisosteric carboxyphenyl motif previously studied. Although an in vitro gel-based TDP1 assay demonstrated reduced TDP1 inhibitory potency compared to imidazo[1,2-*a*]pyrazine **161a**, having a carboxyl group at C-1 position, this work reported the first phosphonic acid-containing small molecule ligands capable of accessing the catalytic pocket of TDP [98].

Recently, Burke, Jr. et al. have modified compounds **160** introducing an oxime linker [99]. The GBB adducts bearing an aminooxy group were initially reacted with 250 aldehydes in microtiter format and nearly 500 oximes were subjected to preliminary in vitro gel-based fluorescence assays. The promising oxime derivatives were further purified and tested for their TDP1 inhibitory activity (Figure 8C). Interestingly, the (*E)-*isomer of oxime **162a** exhibited greater inhibitory potency (IC_50_ = 0.38 μM) compared to (*Z*)-**162a** (5.65 μM). The oxime linker was then replaced with an isosteric triazole (**162b**) or ether (**162c**), obtaining however a slight decrease in TDP1 inhibitory activity. Additionally, the loss of inhibitory potency was observed when the length of the linker was increased. Compounds (*E*)-**162a**, **162b** and **162c** displayed great TDP1 selectivity over TDP2 and showed synergistic effect with TOP1 poison CPT against HCT116 cell lines.

**Figure 9:**
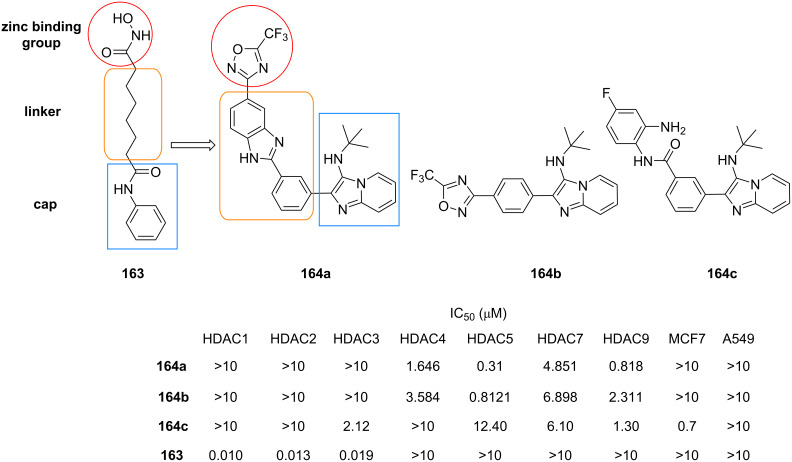
GBB adducts **164a**–**c** as anticancer through in vitro HDACs inhibition assays. Additional cytotoxic assays were conducted against MCF7 and A549 cell lines.

Selective inhibition of histone deacetylases (HDACs) is a promising strategy in the discovery of anticancer agents. There are 18 human HDAC isoforms which can be classified into two different families and four distinct classes based on their structural properties, homology and cellular localization [100]. The first family is composed of zinc-dependent metalloproteins, divided into class I (HADC1, HADC2, HADC3 and HADC8), class IIa (HADC4, HADC5, HADC7, HADC9), class IIb (HADC6, HADC10) and class IV (HADC11); the second family is composed of NAD^+^-dependent proteins and includes class III (Sirt1-7). Al-Tel et al. have developed anticancer agents through selective HDACs inhibition [101]. The HDAC inhibitors were designed based on the FDA-approved HDAC inhibitors for hematologic and solid malignancies treatment, such as vorinostat (SAHA) **163** (Figure 9). Key features were i) a zinc binding moiety (a trifluoromethyloxadiazole (TFMO) or a fluoroaniline), ii) a cap group able to interact with the amino acid residues outside the hydrophobic pocket of HDACs (a GBB-derived imidazopyridine) and iii) a linker between them: to reduce the flexibility observed in vorinostat, a phenylbenzimidazole (**164a**) a phenyl (**164b**) or a benzamide spacer (**164c**) were investigated. The in vitro HDAC inhibition assay demonstrated that the derivatives bearing the TFMO unit **164a** and **164b** were selective toward HDAC5 while derivative **164c** displayed inhibitory activity against HDAC3 and HDAC9. The cytotoxic assay of adducts **164** and vorinostat **163** against breast (MCF7) and lung (A549) cancer cell lines showed that compound **164c** was active against MCF7 cancer cell lines and exhibited non-significant activity on A549 cells, while compounds **164a**, **164b** and **163** were inactive towards both cell lines. To further elucidate the mechanisms of action of compounds **164**, studies on apoptosis and cell cycle progression markers were performed. The activity could be correlated with the downregulation of the antiapoptotic biomarkers such as NF-kB, BCL2, BCL3 and C-MCY as well as the upregulation of proapoptotic proteins such as caspases 3 and 7. Moreover, the expression of cell cycle progression of E2F1, RB1, CDK1, CDK2, CDK4 and CDK6 proteins was downregulated showing that the HDAC inhibitors **164** arrested the cell cycles. Based on the results, the authors concluded that the designed compounds **164** should be effective for the treatment of solid malignancies, such as MCF7.

Dömling et al. reported the synthesis of a 1536 member library of GBB adducts on nanomole scale: 71 isocyanides, 53 aldehydes and 38 cyclic amidines were dispensed in the wells of a 1536 plate using an innovative acoustic dispensing technology (only a subspace of the theoretical 142,994 compounds was synthetized). Although only in 21% of cases the multicomponent adduct was the major product (as determined by MS analysis), the unpurified GBB adducts were screened against the oncogenic protein–protein interaction menin–MLL (Mixed Lineage Leukemia) gene using differential scanning fluorimetry analysis, and several library members were found to be able to bind menin at μM concentrations [102]. This approach has been proposed as an alternative to high throughput screening (HTS) methods. Furthermore, the structural basis of the interactions between the ligands and menin was elucidated by co-crystal structure analysis, highlighting the ability of the imidazopyridine moiety to fit into the binding pocket, generating T-shaped π–π interactions.

#### 4.3 Anti-inflammatory activity

Histone deacetylase 6 (HDAC6) has been associated with the activation of NLRP3 inflammasome, a target for anti-inflammatory therapy. This activation leads to the release of pro-inflammatory cytokines like interleukin IL1B, therefore HDAC6 inhibitors can be potentially used to treat chronic inflammatory diseases [103]. Hansen et al. have prepared 13 imidazo[1,2-*a*]pyridines **166** based on the structure of MAIP-032 **165** [104] and screened their anti-inflammatory activity through the inhibition of the HDAC6 enzyme [105]. Similarly to what reported in Figure 9, the imidazopyridine scaffold served as a cap group, while an hydroxamic acid was used this time as zinc binding group.

**Figure 10:**
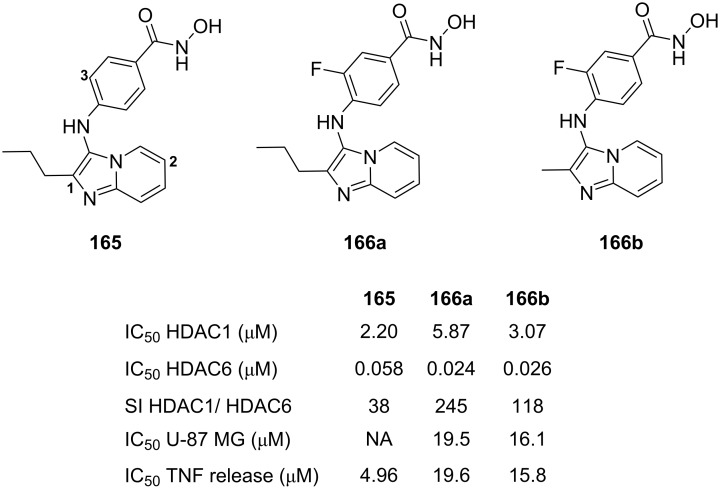
GBB adducts **165**, **166a** and **166b** as anti-inflammatory agents through HDAC6 inhibition; NA = not available. Additional antiproliferative assays against U-87 MG cell lines were carried out.

Molecular docking studies showed that derivatives **166** used their hydroxamic unit to bind Zn^2+^ ions and the secondary amine group to form a hydrogen bond with the serine residue (S531) at the L2-loop. The in vitro assay performed against human HDAC1 and HDAC6 additionally showed that the inhibitory activity against HDAC6 was improved when the C-1 position was decorated with a short or branched alkyl chain while introduction of an alkyl group into the C-2 position increased the inhibition of HDAC1 instead of HDAC6. Introduction of a fluorine atom in position C-3 of the linker improved both HDAC6 inhibitory activity and selectivity towards HDAC1. The results obtained by the most promising candidates **166a** and **166b** are shown in Figure 10.

The antiproliferative assay against glioblastoma U-87 MG cell line revealed that **166a** (IC_50_ = 19.5 μM) and **166b** (IC_50_ = 16.1 μM) were less potent than reference compound vorinostat (IC_50_ = 4.60 μM). In addition, both compounds inhibited the LPS-induced IL1B mRNA expression and TNF release by THP-1 macrophages. The results suggested that **166a** and **166b** could be developed for the treatment of inflammatory diseases driven by NLRP3 inflammasome.

### 5 Novel photophysical properties

It is widely recognized that the GBB-3CR leads to particularly fascinating products in terms of bioactive compounds. However, it is also important to underline that this multicomponent reaction allows a single-step synthesis of complex structures with a significant level of conjugation, hence compounds with potentially interesting photophysical properties, i.e., fluorescence [106]. Despite the evident advantages of this scaffold, examples of fluorescent GBB adducts published before 2019 and used for bio-imaging are limited, if we exclude for example the work by Lavilla et al*.* [49].

**Scheme 46:**
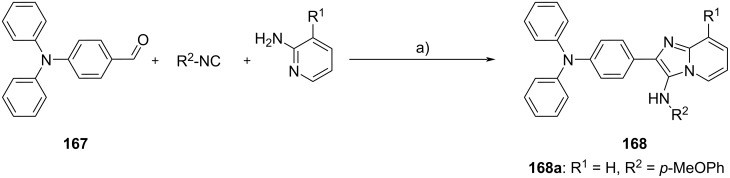
GBB reaction of triphenylamine **167**. Conditions: a) NH_4_Cl (10 mol %), MeOH, 80 °C (MW), 1 h.

Gámez-Montaño et al. reported the synthesis of a series of potentially fluorescent imidazo[1,2-*a*]pyridine-3-amines **168** starting from triphenylamine aldehyde **167**, with a protocol that involved MW heating and NH_4_Cl as catalyst; 16 different compounds have been synthesized in good yields (80–92%) (Scheme 46) [107]. Thanks to an intramolecular charge transfer (ICT) process between the strong electron-donor (triphenylamine) and the acceptor (imidazopyridine), demonstrated by time dependent DFT calculations, the products showed a fluorescence emission from blue to green, achieving a fluorescence quantum yield of 66.2% and a large Stokes shift (6780–9011 cm^−1^). Substitution on the imidazole ring as well as on the 3-amino group could affect the quantum yield and both steric and electronic effects were evaluated, the latter having the greater influence. To prove that these compounds were suitable for bio-imaging applications, they tested compound **168a**, displaying the best quantum yield, as a fluorescent probe in HEK293 and Hela cells (epithelial from human kidney embryo and cervix carcinoma), observing nuclear-specific fluorescence imaging in both cell lines.

**Scheme 47:**
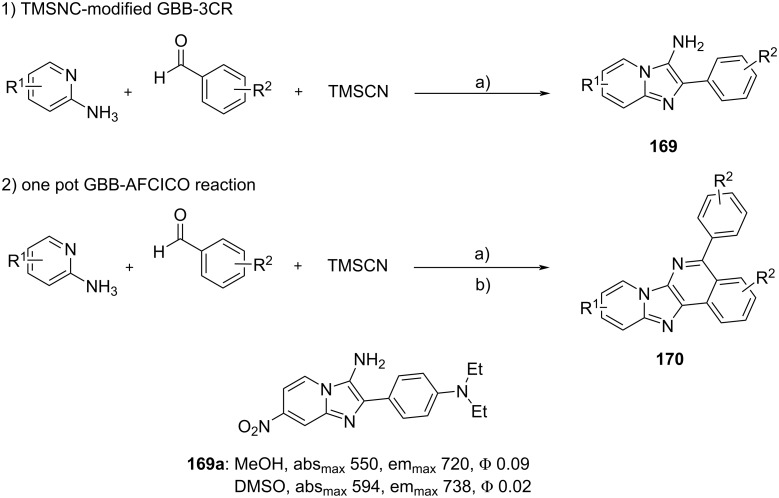
1) Modified GBB-3CR. Conditions: a) TMSCN (1.0 equiv), Sc(OTf)_3_ (0.2 equiv), MeOH, 140 °C (MW), 20 min. 2) One pot synthesis. Conditions: a) TMSCN (1.0 equiv), Sc(OTf)_3_ (0.2 equiv), MeOH, 140 °C (MW), 20 min; b) H_2_SO_4_ (50 equiv), MeOH, 140 °C (MW), 20 min.

In bio-imaging, tuning the absorption and fluorescence emission of probes to higher wavelength is becoming increasingly important as demonstrated by Hulme et al., who published a work about the full spectrum tuning of fluorescent molecules synthesized through MCRs [108]. Specifically, they proposed the synthesis of pyrido[2′,1′:2,3]imidazo[4,5-*c*]isoquinolines and substituted imidazo[1,2-*a*]pyridin-3-amines via the GBB reaction. They studied the tuning of these compounds (achieving emission spectra from 400 to 700+ nm), the structure–photophysical property relationship (SPPR), the solvent effect and they also performed time-dependent DFT (TD-DFT) computations. The syntheses, shown in Scheme 47, involved either a trimethylsilyl cyanide (TMSCN) modified GBB-3CR or, using two equiv of aldehydes, a TMSCN-modified GBB-3CR combined with a one-pot aza-Friedel–Crafts/intramolecular cyclization/oxidation (AFCICO) step. They obtained respectively imidazo[1,2-*a*]pyridin-3-amines **169** and pyrido[2′,1′:2,3]imidazo[4,5-*c*]isoquinolines **170**. Fluorescent analysis showed that better results, in terms of quantum yields and increased wavelength, were obtained with primary amines **169**. It was observed that the most relevant effect for increasing the wavelengths of excitation and fluorescence was the introduction of an electron-withdrawing group on the *para* position of 2-aminopyridine, while electron-donating groups on the aldehyde consistently increased the quantum yield without affecting the fluorescence wavelength: compound **169a** showed an emission maximum above 700 nm, with a quantum yield close to 10% in MeOH. Thanks to TD-DFT computations, these effects were correlated to differing characters of the natural transition orbitals (NTOs).

**Scheme 48:**
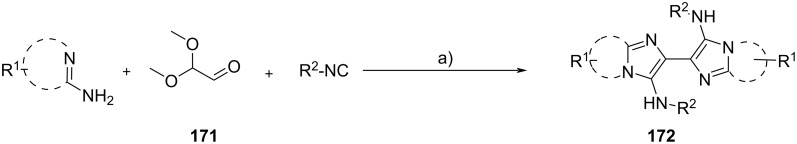
GBB reaction to assemble imidazo-fused heterocycle dimers **172**. Conditions: a) Sc(OTf)_3_ (20 mol %), MeOH, 100 °C (MW), 1 h.

In the work published by Dömling et al., already cited in chapter 2, the GBB-3CR is exploited to obtain imidazo-fused heterocycle dimers. Starting from a new building block, the glyoxal dimethyl acetal, an orthogonal bifunctional monoprotected reactant, they synthesized different structures **172** that have proven to be color-tunable fluorophores [43]. They used the optimized conditions, reported in Scheme 48, to synthesize 37 symmetric GBB dimers **172** and 6 unsymmetric ones (not shown). Investigating the luminescence properties of the obtained compounds, they observed how changing the substituents the emission could be tuned from blue to green and yellow. Exciting at 365 or 370 nm in THF at 25 °C, products **172** with an aromatic amine residue, emitted at 455 nm (blue), while compounds functionalized with alkylamino substituents (*tert*-butylamino or cyclohexylamino groups) shown a red-shifted emission spectrum (respectively 460 and 500 nm). It was also observed that the addition of an electron-donating group on the pyridine, such as a methyl group, led to a slight blue shift (490 nm vs 500 nm). By adding an electron-withdrawing group such as -Br or -CF_3_ on the aromatic amidine, a red shift in the emission spectrum was observed, but associated to a decrease of fluorescence intensity.

A work that deserves to be mentioned also in this chapter is the one of Prasad et al., already reported in chapter 2, for the use of novel sugar-derived building blocks **31** (Scheme 12) [39]. Novel base-modified fluorescent nucleosides are essential to get a deep understanding of structure and function of nucleic acids. Products **31** exhibit fluorescence emission and a good Stokes shift (59–126 nm), therefore seem potentially useful for such applications. Due to the extensive conjugation provided by the imidazopyridine nucleus, these compounds have higher absorption and emission bands compared to parent thymidine.

Other two remarkable works, already presented in previous chapters, are the one presented by Bräse at al. [45] and by Tang et al. [65].

In the first one, the authors synthesized PCP-based imidazo[1,2-*a*]pyridyl-substituted ligands **38** (Scheme 15). These products can be potentially used as spatially-oriented through-space conjugated TADF (thermally activated delayed fluorescence) emitters. Checking their fluorescence properties, it was observed that they are strongly blue fluorescent both in solid state and solution. Changing the partners in the GBB-3CR, the fluorescence could be tuned. The amidine component influenced only slightly the Stokes shift, while strong electron-donating isocyanides could produce a decrease of the Stokes shift and a blue shift. The dimeric PCP adduct, also synthetized in the same study, did not lead to remarkable changes. Fluorescence properties could be modified also by inserting various π-extended or substituted components. A pH-dependency analysis was also carried out, and it was observed that fluorescence decreased in acidic solution, probably due to protonation of the nitrogen atom of the imidazole ring, while an increase of fluorescence and an hypsochromic shift was found in basic solution, possibly due to a destabilization of the excited state.

**Figure 11:**
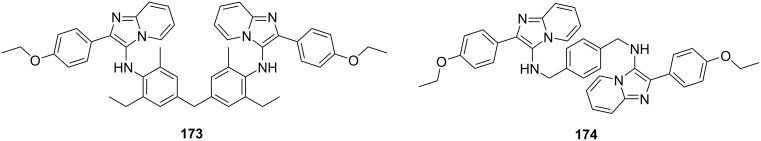
Model compounds **173** and **174**, used to study the acid/base-triggered reversible fluorescence response of polymers **98**.

Tang et al. reported the synthesis of a series of polymers **98** through a transition-metal-free multicomponent polymerization (Scheme 30). These polymers showed acid/base-triggered reversible fluorescence response in solution. In fact, by increasing the amount of HCl, the emission intensity was gradually quenched, while absorption did not drastically change, but, by addition of NaOH, initial fluorescence properties were recovered. This proved that no structure decomposition occurred upon the addition of acid and also that the fluorescence of the polymer could be switched between the “off” state (when an excess amount of HCl was added) and the “on” state (when an equal amount of NaOH was added). The mechanism of fluorescence suppression was revealed by proton NMR studies of model compounds **173** and **174** (Figure 11) in the presence of increasing amounts of HCl in DMSO-*d*_6_: the addition of acid led to the protonation of both the nitrogen atoms of the imidazo[1,2-*a*]pyridine scaffold and the amine moiety, generating a strongly electron-deficient center, resulting in an intramolecular photoinduced electron-transfer (PET) process that quenched the emission [109].

## Conclusion

As demonstrated by the large number and diversity of articles reported in this review, the GBB reaction has now assumed considerable importance in the landscape of multicomponent reactions. As foretold by Dömling et al. in the review published five years ago, new fields of application, bioactive compounds and technologies have been developed in recent years. The use of the GBB reaction in the synthesis of DNA encoded libraries, the first example of a biocatalytic GBB reaction, the exploitation of enabling technologies such as flow chemistry, the preparation of covalent organic frameworks, and the synthesis of tunable bio-imaging agents are just some of the examples discussed in this review. Alongside these more innovative and interdisciplinary features, which make the GBB reaction extremely appealing in emerging fields of application, the incessant search for new catalysts, building blocks and scaffolds confirm the robustness of this reaction in the landscape of organic synthesis. At the same time, the constant search for increasingly potent and selective pharmacologically relevant compounds and the recent interest in the photophysical properties demonstrate the broad spectrum of applicability of GBB adducts. Resuming what was written in the introduction, the GBB reaction undoubtedly occupies a senior position in the family of multicomponent reactions, and the impression of the authors is that, compared with its older relatives, it has much more space for new future developments.

## Data Availability

Data sharing is not applicable as no new data was generated or analyzed in this study.
